# Transcriptome-Based Identification of Highly Similar Odorant-Binding Proteins among Neotropical Stink Bugs and Their Egg Parasitoid

**DOI:** 10.1371/journal.pone.0132286

**Published:** 2015-07-10

**Authors:** Luciana R. Farias, Pedro H. C. Schimmelpfeng, Roberto C. Togawa, Marcos M. C. Costa, Priscila Grynberg, Natália F. Martins, Miguel Borges, Maria Carolina Blassioli-Moraes, Raul A. Laumann, Sônia N. Báo, Débora P. Paula

**Affiliations:** 1 University of Brasília, Campus Universitário Darcy Ribeiro, Brasília-DF, 70910–900, Brazil; 2 Embrapa Genetic Resources and Biotechnology, Parque Estação Biológica, W5 Norte, P.O. Box 02372, Brasília, DF, 70770–917, Brazil; USDA-ARS, UNITED STATES

## Abstract

Olfaction plays a fundamental role in insect survival through resource location and intra and interspecific communications. We used RNA-Seq to analyze transcriptomes for odorant-binding proteins (OBPs) from major stink bug pest species in Brazil, *Euschistus heros*, *Chinavia ubica*, and *Dichelops melacanthus*, and from their egg parasitoid, *Telenomus podisi*. We identified 23 OBPs in *E*. *heros*, 25 OBPs in *C*. *ubica*, 9 OBPs in *D*. *melacanthus*, and 7 OBPs in *T*. *podisi*. The deduced amino acid sequences of the full-length OBPs had low intraspecific similarity, but very high similarity between two pairs of OBPs from *E*. *heros* and *C*. *ubica* (76.4 and 84.0%) and between two pairs of OBPs from the parasitoid and its preferred host *E*. *heros* (82.4 and 88.5%), confirmed by a high similarity of their predicted tertiary structures. The similar pairs of OBPs from *E*. *heros* and *C*. *ubica* may suggest that they have derived from a common ancestor, and retain the same biological function to bind a ligand perceived or produced in both species. The *T*. *podisi* OBPs similar to *E*. *heros* were not orthologous to any known hymenopteran OBPs, and may have evolved independently and converged to the host OBPs, providing a possible basis for the host location of *T*. *podisi* using *E*. *heros* semiochemical cues.

## Introduction

The phytophagous stink bugs, *Euschistus heros* (Fabricius, 1794), *Chinavia ubica* (Rolston, 1983) and *Dichelops melacanthus* (Dallas, 1851) (Hemiptera: Pentatomidae), are responsible for severe damage to important crops in the Neotropical region, mostly soybean in the reproductive stage [1–4]. They locate their host plants through constitutive plant volatiles [5], and signal within their own species through pheromones (e.g. sexual and alarm) [6,7]. These stink bugs also have natural enemies in common, of which the most prominent is the Neotropical egg parasitoid *Telenomus podisi* (Ashmead, 1893) (Hymenoptera: Scelionidae) [8–12]. It locates its stink bug hosts mainly by using olfactory cues, such as plant volatiles released during stink bug oviposition or feeding (synomones) [5,13–16], as well as stink bug pheromones (kairomones) [11,17–28].

In insects, it is well known that olfaction occurs in olfactory sensilla, mostly found in the antenna, through a complex of proteins, in which the odorant binding proteins (OBPs) have been the most studied because of their key role in the first step of olfaction [29,30]. They act as a semiochemical solubilizer, transporter, and ligand-specific selector for mediating the activation of the olfactory receptors enabling insect olfactory perception [30–39]. They were first obtained through biochemical protein isolation from crude antennal extracts (e.g., [40]) and were characterized as small globular water-soluble acidic proteins (about 120–150 amino acids) with a signal peptide in the N-terminal region, six cysteine (Cys) residues in conserved positions, and a predominant alpha-helical secondary structure (e.g., [40,41]). With the application of cDNA libraries (EST-based) and massive DNA parallel sequencing (e.g., [42,43]) OBPs had been found to comprise a multigene family found in 10 different orders, including 32 hemipteran and 43 hymenopteran species (National Center for Biotechnology Information-NCBI). Since then, OBPs have been classified into subfamilies based on the number of conserved Cys: classical OBPs (six), plus-C (more than six and a conserved proline, and according to Ji et al. [44] with a C-terminal extension), minus-C (less than six), and OBP dimers (two complete OBP domains each with six conserved Cys) [37,41,43,45–50].

The pattern of six conserved cysteines (Cys motif) has been used as a signature for OBP identification (e.g., [37,43,48,51,52]). The Cys motif forms three disulphide bridges [47], which, in combination with the amino acid sequence and the physicochemical environment, generate the tertiary structure with a binding pocket that reversibly binds a particular ligand. This OBP domain comprises more than 70% of the amino acid sequence, with a general pattern of low similarity within and among species [41]. Exceptions were observed in some cases intraspecifically, such as in splice variants [53], and interspecifically in some closely related species with known similar ligands [33,54,55]. Such OBP diversity suggests selective binding of odorants in a given species (e.g., [34,55,56]), and may reflect the diversity of chemosensory behavior among the insects [56].

In this work, we analyzed transcriptomes from the main stink bug pest species of soybean in Brazil (*E*. *heros*, *C*. *ubica* and *D*. *melacanthus*), and from one of their main natural enemies, the egg parasitoid *T*. *podisi* to identify and characterize OBP transcripts. Finally, the similarity and phylogeny among these and other available OBPs were analyzed to determine if closely related species share similar OBPs, and if hosts and their natural enemy also share similar OBPs, suggesting they may respond to similar chemical signals/cues.

## Materials and Methods

### Insects

The three stink bugs species, *E*. *heros*, *C*. *ubica* and *D*. *melacanthus*, and the parasitoid *T*. *podisi* were obtained from laboratory colonies in Embrapa Genetic Resources and Biotechnology (Brasília, Distrito Federal, Brazil), and reared according to Borges et al. [57] and Moraes et al. [7] on raw peanuts (*Arachis hypogaea*), soybeans (*Glycine max*), sunflower (*Helianthus annuus*), fresh green beans (*Phaseolus vulgaris*), and water. Insects were kept in 8 L plastic containers (50–60 insects/container) with the food supply renewed three times per week. Unmated male and female adults were placed in separate cages, and after 12 days when the sex pheromone is produced at a consistently high level [58], their antennae were removed using entomological scissors, and immediately used for RNA extraction. *Telenomus podisi* was reared according to Borges et al. [11] in 25 cm^3^ plastic cages, fed with pure honey and provided *E*. *heros* eggs to be used as hosts. When the parasitoids were 20 days-old, they were used for RNA extraction.

### RNA-Seq Construction and Sequencing

A total of 50 pairs of antennae from stink bug males and females (1:1) from each species and 300 *T*. *podisi*, mostly females, were ground in TRIzol reagent (Invitrogen, Life Technologies, Carlsbad, California, USA) and total RNA was obtained according to the method described by Chomczynski and Sacchi [59]. Immediately after extraction, 1 μg of the total RNA of each sample was individually placed in RNAstable tubes (Biomatrica, San Diego, California, USA) and shipped to Beijing Genomics Institute (BGI, China) for sequencing. The integrity/quality of the RNA samples was evaluated using the 2100 Bioanalyzer (Agilent Technologies, Santa Clara, California, USA) and as the RNA Integrity Numbers were ≥ 7.0, the RNA samples were used for RNA-Seq library construction and sequencing using the Illumina GAIIx platform (one lane/sample, 100 bp paired-end).

### *De novo* Transcriptome Assembly and Annotation

The four transcriptome datasets were received in Fastq format. Quality control was performed by Trimmomatic (http://etal.usadellab.org/cms/?page=trimmomatic) using leading 3, trailing 3, sliding window 4:15 and minimum length 36. Before assembly, the high quality paired and unpaired reads were run through VelvetOptimiser 2.2.5 (http://bioinformatics.net.au/software.velvetoptimiser.shtml) to find the best k-mer per dataset in the interval 15 to 47 k-mers. The assembly was first made using Velvet (version 1.2.0.7), and then Oases (version 0.2.0.8) was applied to the output from Velvet (both K-mers). CEGMA software (Core Eukaryotic Genes Mapping Approach) was used (default parameters) to assess the completeness of the transcriptome assemblies, and identify the presence of a core consisting of 248 highly conserved proteins that are found in a wide range of eukaryotes, mostly housekeeping genes, that can be expected to be expressed [60].

To search for related olfaction transcripts, the assembled transcripts (contigs) were annotated using a BLASTx search against a non-redundant protein database from NCBI (E-value≤ 1e-5) and the results were loaded into Blast2GO software version 2.7.0 [61]. The Blast2GO annotation and mapping steps added the gene ontology terms and the transcripts were categorized into biological processes and molecular functions. For each species, the 15 most common biological processes or molecular functions were identified and combined, resulting in a total of 24 biological processes or molecular functions for the four species. Then, the number of annotated sequences in each process or function was normalized by the total number of sequences for each species and the normalized proportions were ranked for statistical analyses with the Cochran-Mantel-Haenszel (CMH) test (Chi-square statistic) on ranks (SAS version 9.3). The species distribution charts of the four species were compared with the Kruskal-Wallis (H) test for independence of ranks.

The putative OBP contigs were screened for open reading frames (ORFs) using the software ORF Finder (http://www.ncbi.nlm.nih.gov/gorf/gorf.html) to identify the coding region. In addition, the OBP domain (SM00708) and gene family (PF01395) were checked by InterProScan 5 [62]. We have named the mined putative OBP ORFs by the first letter of the genus followed by the first three letters of the species name followed by a number in increasing scaffold order. The only exception is for the putative OBP found for *D*. *melacanthus*, which we named using the first two letters of the genus, so that it is not confused with *Drosophila melanogaster* OBPs. The raw sequence transcriptome data were deposited in the Sequence Read Archive (SRA) at DDBJ/EMBL/GenBank database and can be accessed under BioProject PRJNA246320. The assembly results were deposited in the Transcriptome Shotgun Assembly (TSA) and can be accessed as first versions: GBER01000000 for *E*. *heros*, GBFA01000000 for *C*. *ubica*, GBES01000000 for *D*. *melacanthus*, and GBEU01000000 for *T*. *podisi*.

### *In silico* Characterization

The full-length putative OBPs were submitted to a series of *in silico* analyses to predict: the deduced amino acid sequences (Expasy Translate Tool, http://web.expasy.org/translate/); the isoeletric point (pI) and molecular weight (MW) (Compute pI/Mw, http://web.expasy.org/compute_pi/); the signal peptide presence (SignalP 4.1, http://etal.cbs.dtu.dk/services/SignalP/) [63]; as well as tertiary structure and alignment (I-TASSER server 4.2) [64]. To predict the tertiary structure, a query sequence was first threaded by LOMETS through the nr Protein Data Bank (PDB) library to identify structural templates, which were adopted for further structural reassembly, model selection and refinement, following the I-TASSER Suite pipeline [65], which was especially designed for proteins with unknown structure and with low identity (≤25%) with known structures (Table A in S2 File).

### Similarity Analyses

The deduced amino acid sequences of the putative full-length OBPs were aligned with the OBPs from other species available at GenBank using MAFFT 7.017 [66] with the algorithm auto, scoring matrix BLOSUM62, gap opening penalty 1.53, and offset value of 0.123 using Geneious 7.0.5. The OBP sequence orthologs were selected by BLASTx using the criteria of: minimum similarity of E-value< 10e-20 with identity higher than 35% in query coverage of at least 70% for the alignment with the parasitoid putative OBPs, and E-value< 10e-7 with identity higher than 26% in query coverage of at least 77% for the alignment with the target stink bug putative OBPs. We also constructed phylogenetic trees with the mined OBPs from the target stink bugs and the 185 hemipteran OBPs, and with the mined OBPs from the target parasitoid and the 215 hymenopteran OBPs, all available at DDBJ/EMBL/GenBank. First we used the MEGA 6.06 program [67] to find the best Maximum Likelihood (ML) model using the BIC and AICc. Both phylogenetic trees were constructed using the LG+G+I model with 1,000 bootstrap replications, partial deletion of gaps, coverage cutoff 95%, and Nearest-Neighbor Interchange (NNI) ML heuristic method, with strong branch swap filter, to generate an unrooted tree.

## Results and Discussion

### Library Statistics and Transcriptome Assembly, Annotation and Completeness

Transcriptome data of four important economic species in soybean in Brazil were generated and compared for the first time: the phytophagous stink bugs *E*. *heros*, *C*. *ubica* and *D*. *melacanthus*, and their biological control agent the egg parasitoid *T*. *podisi*. The total number of reads obtained after quality control in the stink bug and parasitoid libraries is presented in Table 1, as well as the number and maximum length of contigs. The total number of reads before quality control for the four libraries was similar (data not showed). However, after quality control, the number was uneven, resulting in uneven assembly with 19,697 contigs for *E*. *heros* (3,812 transcripts annotated), 41,860 for *C*. *ubica* (4,594 annotated), 8,647 for *D*. *melacanthus* (1,680 annotated), and 51,106 for *T*. *podisi* (5,540 annotated) (Table B in S2 File). As the RNA samples had similar quality scores (RIN≥ 7.0) and the total number of reads was similar in the four libraries before quality control, the lower number of high quality reads for *D*. *melacanthus* could have occurred due to some bias, possibly in library construction or sequencing. Similarly, regarding the transcriptome completeness assessments, we identified 223 out of 248 constitutive genes indicated by CEGMA for *T*. *podisi*, i.e. 89.9% completeness, while for the stink bugs it was 50.0% for *C*. *ubica*, 44.35% for *E*. *heros*, and 14.9% for *D*. *melacanthus* (Table C in S2 File). One factor that may have influenced the completeness was that the parasitoid *T*. *podisi* transcriptome was extracted from the whole body, while the transcriptomes from the stinkbugs were from a specialized tissue (antennae). The uneven number of annotated contigs obtained from the four target species reflected both the BLASTx results and annotation process: 5,540 annotations out of 22,926 BLASTx results for *T*. *podisi*, 4,594 annotations out of 7,370 for *C*. *ubica*, 3,812 out of 5,142 for *E*. *heros*, and 1,680 out of 2,346 for *D*. *melacanthus*. The predicted olfaction related transcripts are indicated in Tables D to G in S2 File. The total number of putative olfaction related transcripts was similar for the stink bugs *E*. *heros* (23 OBPs, with four full-length) and *C*. *ubica* (25 OBPs, with four full-length), but lower for the stink bug *D*. *melacanthus* (9 OBPs, with one full-length) and for the parasitoid *T*. *podisi* (7 OBPs, with three full-length). We previously identified two other putative full-length *E*. *heros* OBPs (EherOBP1 and EherOBP2, GenBank codes ADJ18275.1 and ADO24165.1, respectively) [68] through antennal cDNA library screening, so the total number of putative OBPs for *E*. *heros* is actually 25, the same as found for *C*. *ubica*. The lower number of putative olfaction transcripts from *D*. *melacanthus* compared to the other stink bugs might be related to its lower transcriptome coverage, although it could also be related to a biological reason. Studies on *D*. *melacanthus* have shown that they produce a similar blend of defensive compounds identified in other stink bugs [58,69,70], but there is currently no report identifying its sex or aggregation pheromone. It is not yet possible to determine if this species has or does not have similar chemical communication as other stink bugs, i.e, this species may also use non-chemical cues to locate host and partners, such as vibrational or visual signals, which could explain why they have fewer putative OBPs [7]. Regardless, the number of OBPs identified could have been higher by using a higher performance sequencing or increasing the number of lanes used.

**Table 1:** Number of reads obtained from the RNA-Seq libraries and Illumina GAIIx sequencing for the stink bug antennae and whole-body parasitoid after quality control, and number of contigs assembled for each species. The minimum contig length was 300 bp for all samples. QC: quality control.

		Number of reads			Contig		
Species	GB of data	Before QC	Paired after QC	Unpaired after QC	Total number	Maximum length (bp)
*E*. *heros*	11.39	46,099,954	4,891,510	5,783,001	19,697	6,867
*C*. *ubica*	10.30	41,693,722	9,194,804	7,554,025	41,860	12,263
*D*. *melacanthus*	12.42	50,287,824	3,254,542	4,921,482	8,647	4,229
*T*. *podisi*	10.37	41,960,524	10,519,096	11,282,464	51,106	12,696

Gu et al. [71] constructed cDNA libraries from the male and female antennae of the lucerne plant bug, *Adelphocoris lineolatus* (Hemiptera: Miridae), and selecting 2,915 ESTs they obtained 1,423 unigenes (with 215 contigs) with an average length of 532 bp and 549 bp for ESTs from the male and the female libraries, respectively, from which were annotated 14 putative OBPs. In the antennal cDNA library from the green plant bug *Apolygus lucorum* (Hemiptera: Miridae), Ji et al. [44] obtained 5,018 ESTs, which assembled into 3,881 unigenes (with 783 contigs) with an average length of 144 bp, from which were annotated 12 putative OBPs that also had a molecular function of putative binding proteins.

The number of putative OBPs found in the antennae of the stink bugs *E*. *heros* and *C*. *ubica* was higher than that found in the lucerne and green plant bugs, however, the plant bug studies produced the transcriptome through Sanger sequencing instead of high throughput sequencing and had a much smaller number of unigenes/contigs from which to search for putative OBP transcripts. The heterogeneity of OBPs mined from the Neotropical stink bugs *E*. *heros* and *C*. *ubica* was also higher than that identified for other hemipterans, including the aphids *Acyrthosiphon pisum*, with 15 putative OBPs identified through its genome sequence [41], and *Aphis gossypii*, with 10 OBPs identified using a high throughput sequencing platform (from total of 54,547 unigenes) [72], the human body louse, with five genes obtained from its genome project [73], and the white-backed planthopper *Sogatella furcifera*, with 12 OBPs obtained from a transcriptome analysis [74]. The only exception was the tarnished plant bug *Lygus lineolaris*, from which 33 putative OBPs were found using a high throughput sequencing platform; however, instead of a single life stage, the researchers sequenced all developmental stages to generate the libraries [53]. No comparison was possible with other Pentatomidae species because we were the first group to identify putative OBPs in this family [68].

In the parasitoid wasp, *Cotesia vestalis*, using transcriptome pyrosequencing of antennae, Nishimura et al. [75] obtained 17,238 contigs (average size 549 bp) and 31,921 singletons, which had a similar species distribution as that found for the target parasitoid *T*. *podisi*. They annotated 22 putative OBPs, a higher number of OBPs than mined from the whole body of *T*. *podisi*. The number of OBPs from *T*. *podisi* was also smaller than that obtained from genome analyses of other hymenoptera, e.g., 12 putative OBPs mined from the fire ant *Solenopsis invicta* [76], 21 from *Apis mellifera* [48], and 90 from the parasitoid *Nasonia vitripennis* [50]. Despite the high transcriptome coverage of *T*. *podisi* (51,106 contigs), the smaller number of OBPs mined from it compared to these other hymenopteran species might be related to having analyzed the whole body, instead of a tissue specialized in olfaction, such as the antenna. However, in the solitary bee *Osmia cornuta*, Yin et al. [77] annotated only six putative OBPs from a transcriptome analysis of the antennae using high throughput sequencing, which assembled 85,143 contigs (average length of 914.5 bp).

### Transcriptome Comparisons

**Fig 1:**
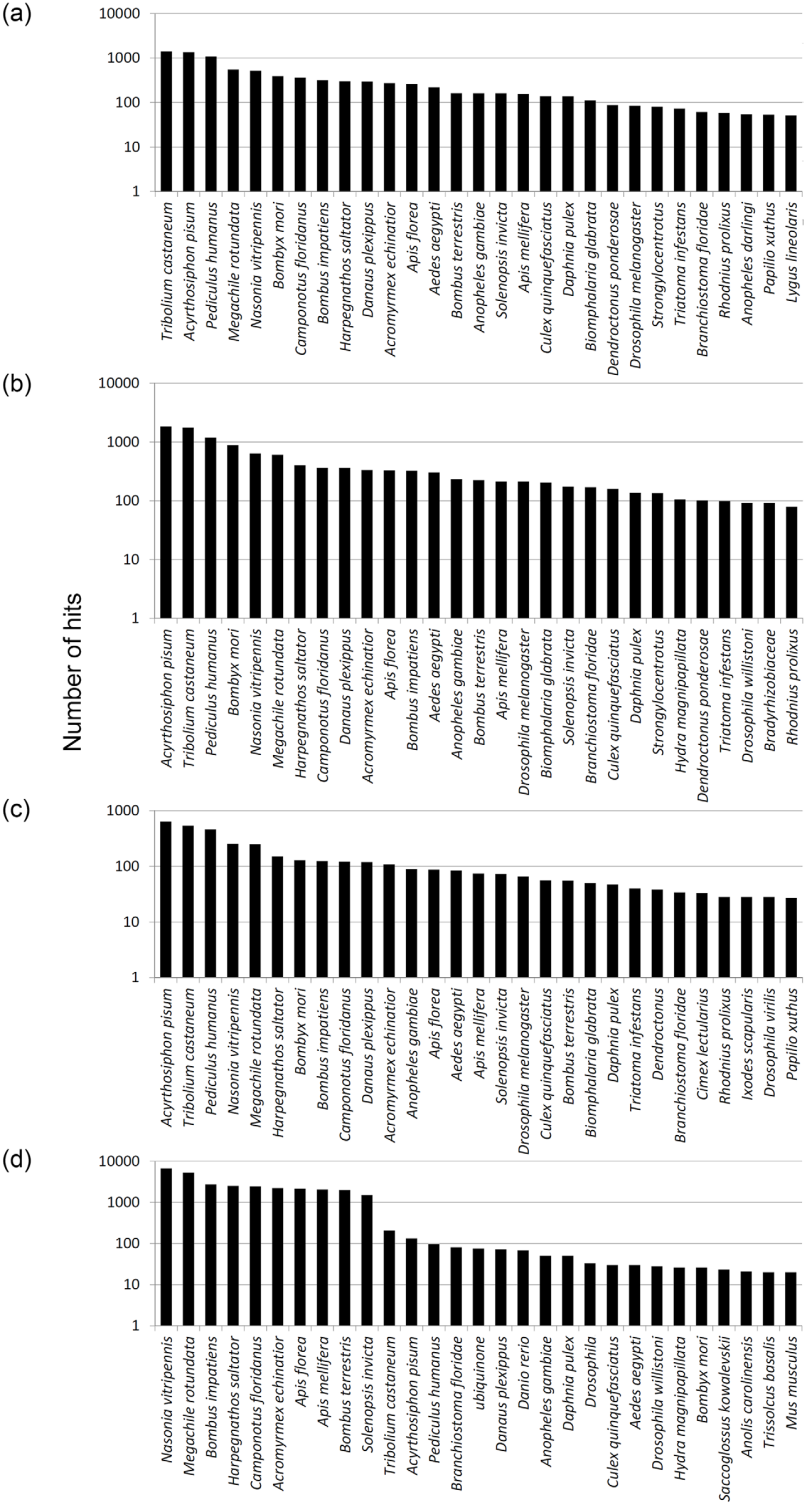
Distribution of species with the highest number of hits with similarity to contigs from antennae of 12 day-old virgin adults of stink bugs and whole body of 20 day-old parasitoid: (a) *E*. *heros* (n = 11,035 top BLASTx hits, 10.8% total BLASTx hits), (b) *C*. *ubica* (n = 14,869, 11.02%), (c) *D*. *melacanthus* (n = 4,789, 11.1%) and (d) *T*. *podisi* (n = 31,619, 10.4%).

The BLASTx search using the transcripts reconstituted from the transcriptome of the target stink bugs and the parasitoid matched homologous sequences in other species (Fig 1, BLASTx top 5 hits in S1 File). Of the 40 species with the highest number of matches, 21 matched with all four target species, and these were retained for statistical analysis. The 19 species that did not have matches with all four target species were Coleoptera (*Dendroctonus ponderosae*), Diptera (*Anopheles darling*, *Drosophila virilis*, *Dr*. *willistoni*), Hemiptera (*Cimex lectularius*, *L*. *lineolaris*, *Rhodnius prolixus*, *Triatoma infestans*), Hymenoptera (*Trissolcus basalis*), Ixodida (*Ixodes scapularis*), Lepidoptera (*Papilio xuthus*), Chordata (*Anolis carolinensis*, *Danio rerio*, *Mus musculus*), Hemichordata (*Saccoglossus kowalevskii*), Echinodermata (*Strongylocentrotus purpuratus*), Mollusca (*Biomphalaria glabrata*), Cnidaria (*Hydra magnipapillata*), and Proteobacteria (*Bradyrhizobiaceae bacterium*). The Hemiptera species matched with the target stink bugs, whereas *T*. *podisi* had higher matches with hymenopteran species, especially with the parasitoid *N*. *vitripennis*, and with the egg parasitoid *Tr*. *basalis*.

The retained 21 match species were ranked among the three target stink bugs and parasitoid data (Table 2). The distribution of species matches was significantly different among the three target stink bug hosts and their parasitoid (H = 146.803, df = 60, P< 0.0001). As expected, most matches were with species in the same order as the target species. For the stink bugs the main species were Hemiptera, and for the parasitoid the main species were Hymenoptera. Six species were significantly different among the four target species. *Acyrthosiphon pisum*, *Tri*. *castaneum*, *P*. *humanus*, and *Bombyx mori* were similarly ranked among the three stink bugs, but ranked significantly lower in the parasitoid. The first three species were also the most similar to the antennal transcriptome of the 3 day-old adult hemipteran *A*. *lineolatus* (Miridae) [71]. *Bombus terrestris* and *N*. *vitripennis* were significantly higher ranked in the parasitoid compared to the stink bugs, and the parasitoid top-hits species distribution was similar to that found for the antennal transcriptome of 24 h adult wasps *Co*. *vestalis* (Braconidae) [75]. Based on this initial result, data were analyzed for the three stink bugs only. There was no difference in match species among these three species (H = 5.994, df = 40, P = 1.000).

**Table 2:** Match species, rank of the highest normalized number of hits for the target stink bugs and parasitoid, Kruskal-Wallis H value (H) and type I error probability (P). Match species in bold are significantly different among the target stink bugs and parasitoid.

Match species	*E*. *heros*	*C*. *ubica*	*D*. *melancanthus*	*T*. *podisi*	H	P
Hemiptera						
***Acyrthosiphon pisum***	7	4	2	75	27.504	<0.0001
Hymenoptera						
*Acromyrmex echinatior*	40	42	43	16	5.259	0.1538
*Apis florea*	41	44	49	18	5.434	0.1427
*Apis mellifera*	60	55.5	52	20	7.749	0.0515
*Bombus impatiens*	30	45	35	12	6.175	0.1034
***Bombus terrestris***	57	53	67	22	8.050	0.0450
*Camponotus floridanus*	28	38	36	15	3.909	0.2714
*Harpegnathos saltator*	33	31	29	13	3.540	0.3156
*Megachile rotundata*	21	25	19	3	5.928	0.1151
***Nasonia vitripennis***	23	24	17	1	7.910	0.0479
*Solenopsis invicta*	58.5	64	54	26	6.321	0.0970
Diptera						
*Aedes aegypti*	47	46	50	82.5	1.558	0.6689
*Anopheles gambiae*	58.5	51	48	79.5	0.893	0.8271
*Culex quinquefasciatus*	62	68	65	82.5	0.105	0.9912
*Drosophila melanogaster*	71	55.5	61	81	0.525	0.9133
Coleoptera						
***Tribolium castaneum***	5	6	8	74	22.884	<0.0001
Lepidoptera						
***Bombyx mori***	27	14	32	84	11.053	0.0114
*Danaus plexippus*	34	39	37	78	3.250	0.3546
Phthiraptera						
***Pediculus humanus***	10	11	9	76	18.487	0.0003
Cladocera (Crustacea)						
*Daphnia pulex*	63	70	69	79.5	0.086	0.9934
Cladocera (Crustacea)						
*Branchiostoma floridae*	73	66	72	77	0.181	0.9805

Overall, comparing the BLASTx results between the stink bug species and the parasitoid, the number of hits found for the parasitoid was more than twice that for the stink bug species. This may reflect the difference in the starting number of contigs (more from *T*. *podisi*) or indicate that the transcriptome of a specific tissue (antenna) may have poorer representation in the public databases. The list of matching species should be considered carefully, as it could be related to the proportion and representation of species in the public databases (and the particular physiologic condition of the insects from which the transcriptomes were obtained) rather than having a phylogenetic meaning.

**Fig 2:**
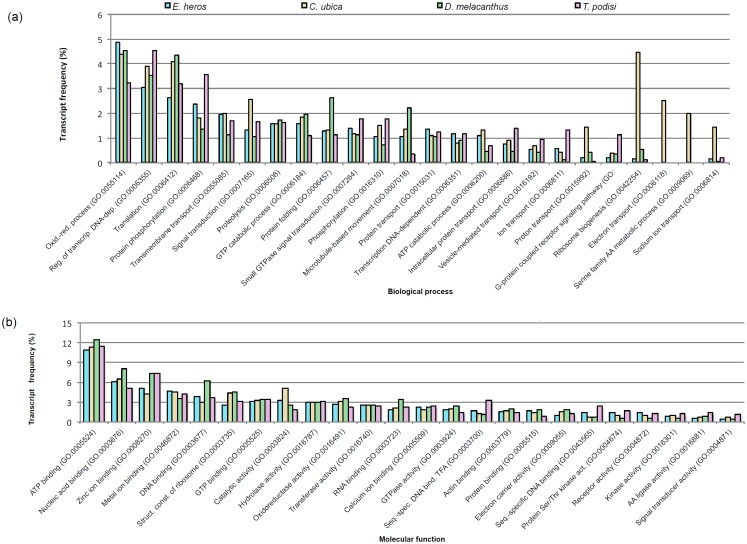
Most frequent biological processes (a) and molecular functions (b) categories from the antennae of 12 day-old virgin adults of stink bugs and whole body of 20 day-old parasitoid. The number of transcripts for a particular GO term (at lower hierarchy level) in each category was normalized by the total number of classified transcripts.

The relative abundance of the transcripts was also analyzed based on Gene Ontology (GO) classifications for biological process and molecular function (Fig 2). Some of the most common biological processes differed quantitatively among the target species (CMH = 58.34, df = 21, P*<* 0.0001). The three most common of these were oxidation-reduction processes, regulation of transcription DNA-dependent, and translation. Of the 24 most common biological processes in the four target species, *C*. *ubica* had the highest richness and differed from the others in ribosome biogenesis, electron transport, serine family amino acid metabolic process, signal transduction, sodium ion transport and proton transport. *Telenomus podisi* differed from the others in ion transport, and all four species differed from each other in microtubule-based movement.

The molecular functions had a rich representation of binding activities (such as to ATP, nucleic acids and ions). The most common molecular functions also differed quantitatively among the species (CMH = 81.67, df = 21, P*<* 0.0001). Of the 24 most common molecular functions in the four target species, *D*. *melacanthus* differed from the others in RNA binding; *T*. *podisi* differed from the others in catalytic activity, and sequence-specific DNA binding (GO:0043565 and GO:0003700). All four species differed from each other in protein serine/threonine kinase activity.

**Fig 3:**
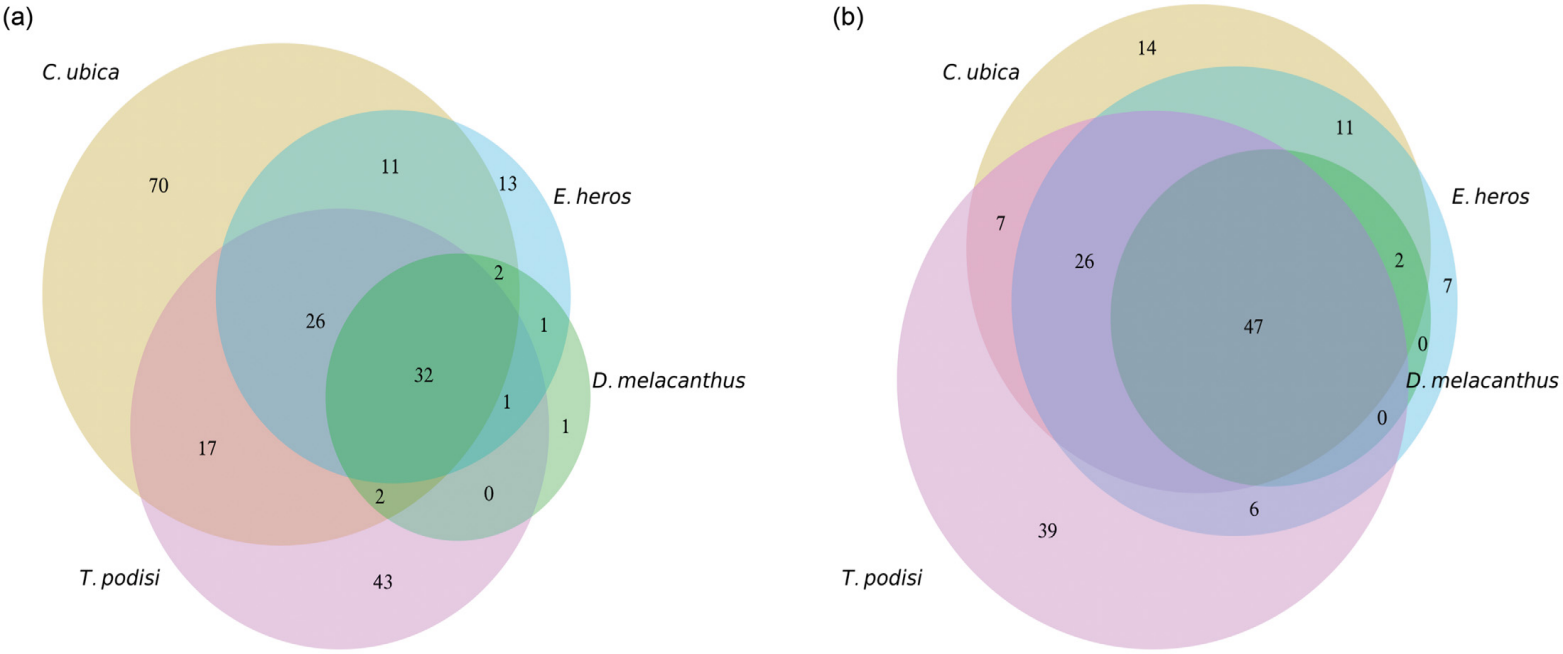
Number of GO categories exclusive and common to each species: (a) biological process, and (b) molecular function. A species was considered to have the category if it had more than 10 transcripts. The radii of the circles are proportional to the number of categories.

The biological processes diverged more among the target species than the molecular functions, as can be observed in Fig 3. More biological processes were identified in the antennae of *C*. *ubica* (n = 160, 70 exclusive), than in *T*. *podisi* (n = 121, 43 exclusive), *E*. *heros* (n = 87, 13 exclusive) and *D*. *melacanthus* (n = 39, 1 exclusive). In total, 32 biological processes were shared among the four species, but only two were exclusive to the three stink bug species. More molecular functions were identified in the whole body of *T*. *podisi* (n = 121, 39 exclusive), than in the stink bugs *C*. *ubica* (n = 107, 14 exclusive), *E*. *heros* (n = 99, 7 exclusive), and *D*. *melacanthus* (n = 49, none exclusive). The stink bugs shared more molecular functions among themselves than with the parasitoid *T*. *podisi*.

Overall, the transcriptome composition of the stink bugs and the parasitoid was similar to other hemipteran and hymenopteran species. In the antenna of 3 day-old adult hemipterans *A*. *lineolatus* [71] and *Ap*. *lucorum* [44], the relative abundance of the transcripts also had higher representation of binding and catalytic activity in the molecular function ontology, and cellular process in the biological process ontology. In the 24 h emerged adult wasps *Co*. *vestalis*, the genes expressed in the antennae were also mostly associated with molecular binding activity or categorized as catalysts [75].

### *In silico* Characterization

**Fig 4:**
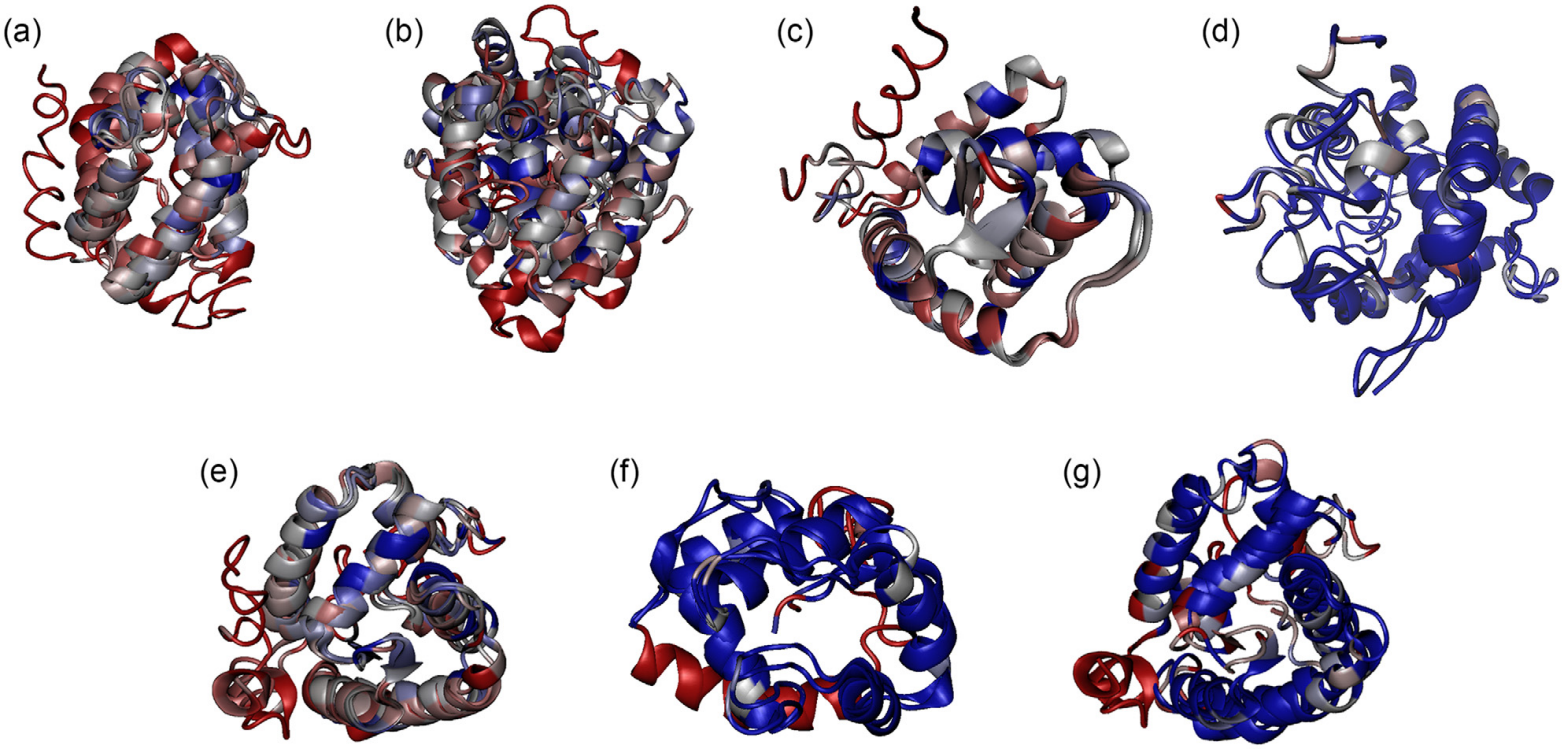
Alignment of the predicted tertiary structures of the full-length putative OBPs from the stink bugs *E*. *heros*, *C*. *ubica* and *D*. *melacanthus* and from the parasitoid *T*. *podisi*. Blue shows structural similarities and red shows dissimilarities according to the matrix Blosum60 for: (a) within EherOBPs; (b) within CubiOBPs; (c) between EherOBP3 and CubiOBP3; (d) between EherOBP6 and CubiOBP1; (e) within TpodOBPs; (f) between TpodOBP1 and EherOBP2; (g) between TpodOBP2 and EherOBP1. The structures were generated by I-TASSER server 4.2 and were oriented with the N-terminal to the right side.

The full-length putative OBPs (Table 3) revealed OBPs with physicochemical properties similar to other OBPs [31,33,78]. They are small (136–212 residues and 14,627.8–23,783.1 Da), have an amino terminal signal peptide (from position 1 to around 20) followed by the OBP domain until a few residues prior to the carboxyl terminal end, and have an acidic pI< 6, except for DimelOBP1 with a pI of 6.57 and TpodOBP3 with an unusual basic pI of 9.41. The predicted tertiary structures had five to seven alpha-helixes (Fig 4), held together by three disulfide bridges between Cys1-Cys3, Cys2-Cys5, and Cys4-Cys6, as expected for an OBP structure [38].

**Table 3:** Physico-chemical predictions for the putative full-length OBPs obtained from the target stink bugs (*E*. *heros*, *C*. *ubica* and *D*. *melacanthus*) and parasitoid (*T*. *podisi*). AA: Amino acid residue number; pI: Isoelectric point; MW: molecular weight (Da); Cys: cysteine; SignalP: signal peptide amino acid location. InterPro domains IPR023316 and SM00708, and families IPR006170 and PF01395. Unless otherwise indicated the “Conserved Cys” is six classical.

OBP	Accession code	AA	pI	MW	Conserved Cys	SignalP	Domain PBP/GOBP
EherOBP3	KM213228	155	4.79	17,529.8	6	1–19	PBP/GOBP 18–130 and PBP 28–131
EherOBP4	KM213229	151	5.79	17,033.8	6	1–19	PBP/GOBP 17–135 and PBP 31–135
EherOBP5	KM213230	172	5.37	18,967.1	6	1–15	26–167
EherOBP6	KM213231	211	5.44	23,669.0	12 (Plus-C)	1–19	48–135
CubiOBP1	KM213232	212	5.43	23,783.1	12 (Plus-C)	1–19	47–136
CubiOBP2	KM213233	143	5.57	15,943.5	6	1–20	PBP/GOBP 23–130 and PBP 28–131
CubiOBP3	KM213234	140	4.59	15,406.4	6	1–19	PBP/GOBP 18–127 and PBP 28–131
CubiOBP4	KM213235	152	5.29	17,031.8	6	1–15	PBP/GOBP 15–137 and PBP 32–137
DimelOBP1	KM213236	141	6.57	15,710.5	5 (minus-C)	1–23	PBP/GOBP 37–137 and PBP 21–127
TpodOBP1	KM213272	136	4.46	14,627.8	6	1–18	GOBP/PBP 15–129 and PBP 32–129
TpodOBP2	KM213238	148	5.02	16,547.1	6	1–19	GOBP 30–136 and PBP 19–131
TpodOBP3	KM213239	143	9.41	16,592.5	6	1–19	GOBP/PBP 38–138 and PBP 37–137

Regarding the C-pattern, the putative OBPs from the target stink bugs and parasitoid had six cysteines (Cys) in conserved positions, as in a classical OBP, except for DimelOBP1 with five Cys (minus C6), which would be classified as a minus-C OBP [43]. In this work, all putative OBPs have the strictly conserved three residues between C2-C3, and most of them (9 of 14) have the expected Cys motif-pattern for Hemiptera (C1-X_22-32_-C2-X_3_-C3-X_36-46_-C4-X_8-14_-C5-X_8_-C6) [52], except for EherOBP1 and EherOBP5 with C1-X_41_-C2, EherOBP5 and EherOBP6 and CubiOBP1 with C4-X_22_-C5, EherOBP6 and CubiOBP1 with C5-X_9_-C6, and TpodOBP2 with C3-X_46_-C4. In addition, EherOBP6 and CubiOBP1 have additional conserved Cys: C1a, C1b, C1c, C6a, C6b and C6c, a proline (P) after the C6a, and an extended C-terminal end. These characteristics indicate that EherOBP6 and CubiOBP1 are OBP Plus-C [43]. Therefore, of the six putative OBPs found for *E*. *heros*, five are classical OBPs and one is a Plus-C OBP, and of the four found for *C*. *ubica*, three are classical OBPs and one is a Plus-C OBP.

The number of Plus-C OBPs found was less than that from other Hemiptera species, e.g., two from *A*. *lineolatus* [71]; four-six from *Ap*. *lucorum* [44,71]; 12 from *L*. *lineolaris* [53]; two from pea aphid *A*. *pisum* [41] and three from *S*. *furcifera* [74]. For the parasitoid, all three full-length putative OBPs had the classical C-pattern, no other OBP category was found, similar to the parasitoid *N*. *vitripennis*, with a large number (90) of putative OBPs identified [50], and for *Co*. *vestalis* [75].

According to Xu et al. [52], in most insects the typical OBP motif C-pattern has three amino acids between the second (C2) and third (C3) Cys, and eight residues between the fifth (C5) and sixth (C6) Cys. They hypothesized the existence of an order-specific Cys motif-pattern for OBPs, such that in Hemiptera the pattern would be C1-X_22-32_-C2-X_3_-C3-X_36-46_-C4-X_8-14_-C5-X_8_-C6, and in Hymenoptera it would be C1-X_23-35_-C2-X_3_-C3-X_27-45_-C4-X_7-14_-C5-X_8_-C6, where X stands for any amino acid. Of the 14 putative OBPs mined, four of the stink bug OBPs and one of the parasitoid OBPs do not completely fit this hypothesized order-specific Cys motif-pattern, although all putative OBPs have the strictly conserved three residues between C2-C3.

### OBP Similarity

**Fig 5:**
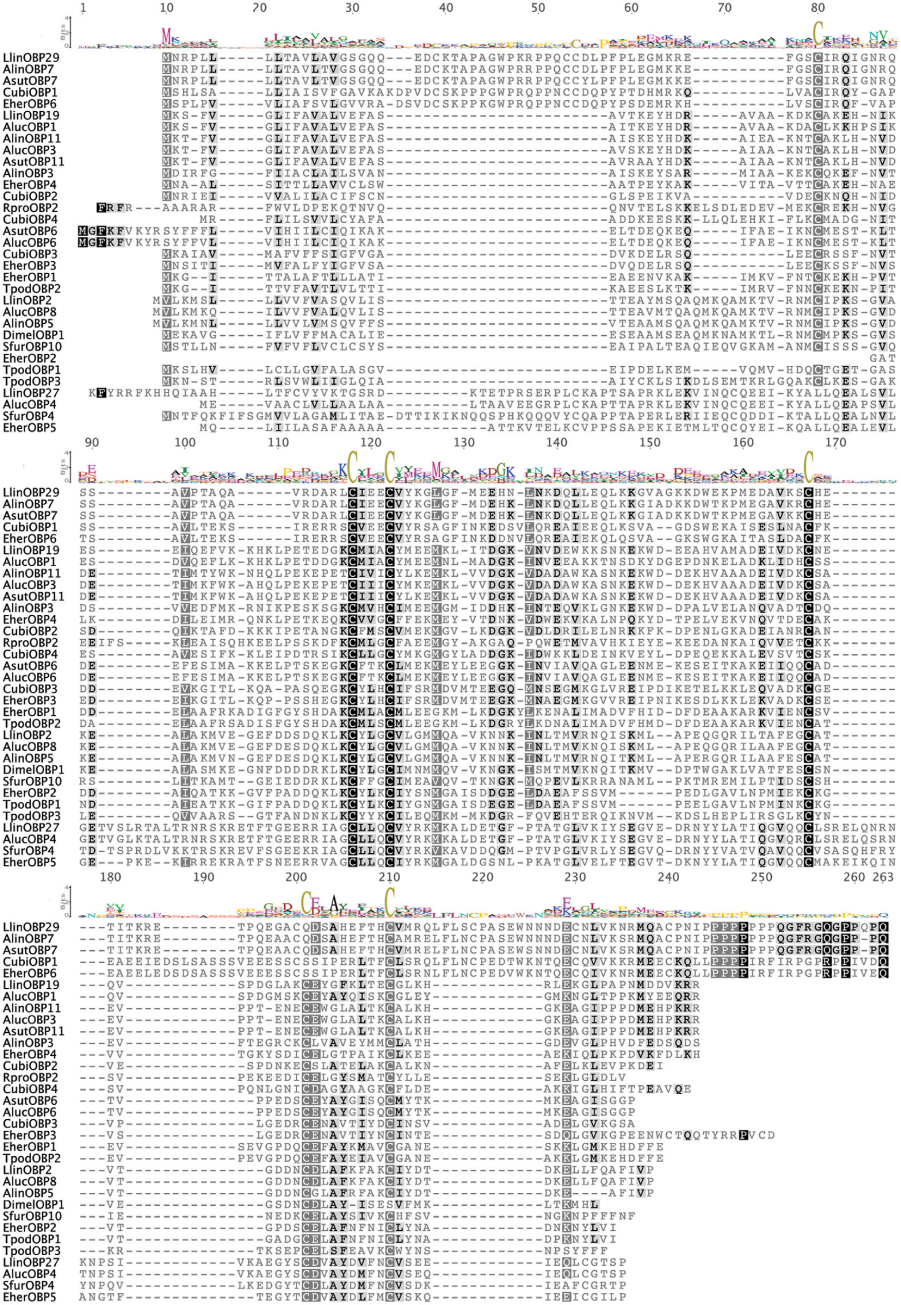
Alignment of the deduced amino acid sequences of OBPs from the stink bugs and other Hemiptera obtained from GenBank by BLASTx. Similarity is scored by matrix Blosum62 where the black color indicates 100% identity, darker grey 100% > identity ≥ 80%, lighter grey 80% > identity ≥ 60% and white color identity <60%. The sequence logo is at the top of the alignment. The amino acid percentage identity matrix is presented in Table H in S2 File. The conserved Cys are indicated by sequence logo. The species names are abbreviated with four letters, and their full names with all accession numbers of the OBP amino acid sequences are provided in Table I in S2 File.

**Fig 6:**
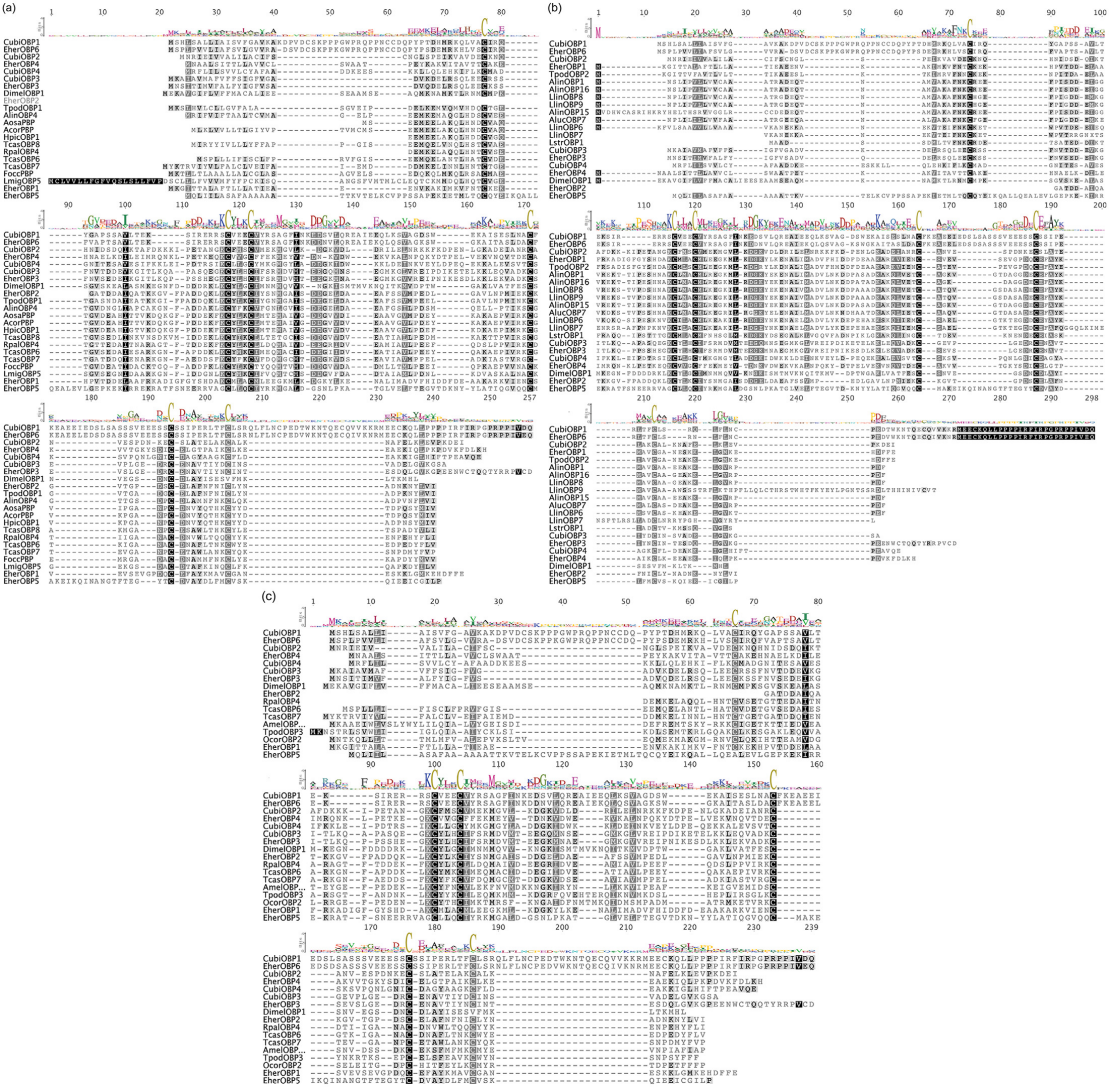
Alignment of the deduced amino acid sequences of OBPs from the parasitoid *T*. *podisi* and the most similar OBPs obtained from GenBank by BLASTx: (a) TpodOBP1; (b) TpodOBP2; (c) TpodOBP3. Similarity is scored by matrix Blosum62 where the black color indicates 100% identity, darker grey 100% > identity ≥ 80%, lighter grey 80% > identity ≥ 60% and white color identity <60%. The sequence logo is at the top of the alignment. The amino acid percentage identity matrix is presented in Table H in S2 File. The conserved Cys are indicated by sequence logo. The species names are abbreviated with four letters, and their full names with all accession numbers of the OBP amino acid sequences are provided in Table I in S2 File.

The similarity analysis of the mined hypothetical OBPs from the target stink bugs and parasitoid (Fig 4) with other orthologous OBPs from different insect species is represented in Figs 5 and 6 and in Table H in S2 File. The predicted tertiary structure of the stinkbug and parasitoid full-length putative OBPs are individually presented in Figure A in S2 File. In all the alignments the most conserved regions were around the conserved Cys. The sequence similarity among EherOBPs ranged from 5.1 to 25.2%, among CubiOBPs from 9.3 to 25.3%, and among TpodOBPs from 16.3 to 27.5%.

The highest sequence similarities were obtained from four interspecific comparisons. *Euschistus heros* and *C*. *ubica* had two cases of high similarity across their OBPs: between EherOBP6 and CubiOBP1 with 84% similarity, and between EherOBP3 and CubiOBP3 with 76.4% similarity. The most interesting findings of this work were the high similarities of OBPs between the preferred stink bug host and its natural enemy: TpodOBP1 & EherOBP2 (88.5%) and TpodOBP2 & EherOBP1 (82.4%). TpodOBP1 also had similarity to OBPs from the Coleoptera, Hemiptera, Orthoptera, and Thysanoptera (ranging from 29.4 to 49.3%), and TpodOBP2 only with hemipteran OBPs (ranging from 28.6 to 44.2%). TpodOBP3 was the only one similar to hymenopteran OBPs, although at no more than 30% similarity. The alignment of the predicted tertiary structure between these four pairs also presented high similarity, especially among the α-helixes (Fig 4). As expected, the loops were the most variable regions.

Previous studies have generally found low OBP similarities among species, typically < 40% [71], with a few exceptions among related species. Lepidopteran Pheromone Binding Proteins (PBPs) and general OBPs have 31–91% and 47–100% similarities respectively [33]. The OBPs from 12 aphid species all cluster to 10 groups with 81.2% similarity within groups compared to an overall similarity of only 20.0% [72]. High interspecific similarity was not common within the Heteroptera and only two pairs of mirid OBPs had high similarity, 89% and 74% [44,71]. The high similarities observed between EherOBP6 & CubiOBP1 and EherOBP3 & CubiOBP3 are consistent with these reports. Only Yin et al. [77] report similarities of OBPs from insects of different families, and the highest observed similarity was 65%. The observed similarity across insect orders between TpodOBP1 & EherOBP2, and TpodOBP2 & EherOBP1 was much higher than previously reported for phylogenetically unrelated species.

**Fig 7:**
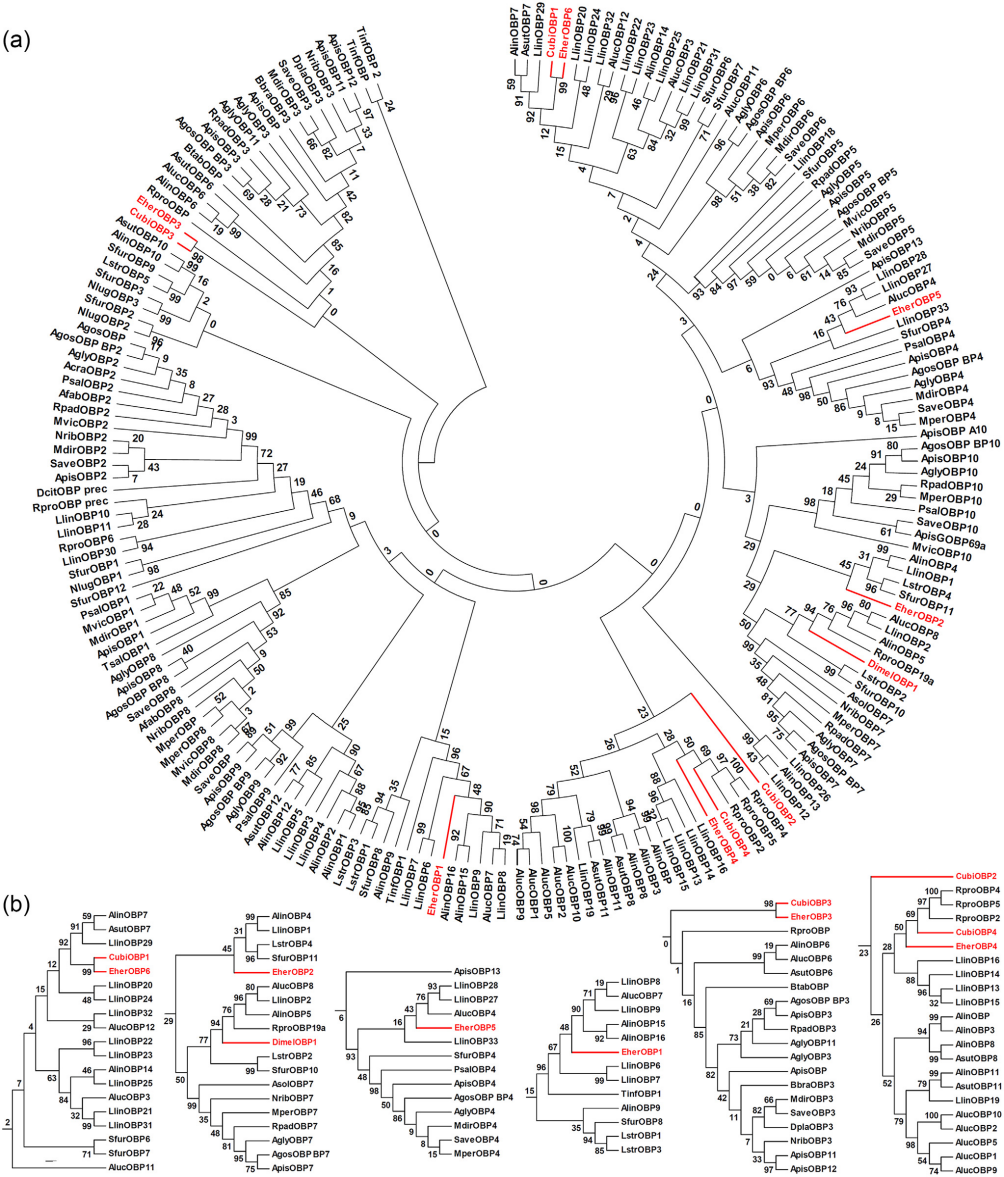
Phylogenetic relationships of: (a) target stink bug putative OBPs (in red) and putative 185 other hemipteran OBPs; (b) Detailed relationships of the putative EherOBPs, CubiOBPs, and DimelOBP. The trees were constructed with MEGA 6.06 using a LG+G+I model. Values indicated at the nodes are bootstrap values based on 1,000 replicates presented with 50% cut-off bootstrap value for (a) and no cut-off for (b). The species names are abbreviated with four letters, and their full names with all accession numbers of the OBP amino acid sequences are provided in Table I in S2 File.

The phylogenic analysis (Fig 7) for the target stink bug OBPs with the other 185 hemipteran sequences from 28 species confirmed the intraspecific divergence of their putative OBPs. The OBP phylogeny did not correspond to the known hemipteran phylogeny. The intraspecific OBPs did not form a single clade, and for both *E*. *heros* and *C*. *ubica*, their OBPs mapped to different clades. At the same time, the phylogeny showed the convergence or shared derived character of the two pairs of similar putative OBPs across *E*. *heros* and *C*. *ubica*. EherOBP6 and CubiOBP1 were sisters with high (99) bootstrap support. They had three mirid OBPs as their closest outgroup with high (92) bootstrap support, but the next closest outgroup was unclear (bootstrap support 12). EherOBP3 and CubiOBP3 were sisters with high (98) bootstrap support, but they were not clearly linked to any outgroup (bootstrap support 0).

The phylogenetic relations of the other stink bug OBPs revealed two patterns. The first was monophyly with early divergence from other OBPs. EherOBP1 was monophyletic with one reduviid and 7 mirid OPBs with high (96) bootstrap support (Fig 7B), EherOBP5 was monophyletic with four mirid, one delphacid, and seven aphidid OBPs (bootstrap support 93), and DimelOBP1 was monophyletic with three mirid and one reduviid OBPs (bootstrap support 94). The EherOBP6 & CubiOBP1 pair mentioned above was also a part of a monophyletic group of three mirids (bootstrap support 92). As the database is enriched with more species of Heteroptera, these clades are likely to remain monophyletic, but the phylogenetic pattern within the clade will become clarified.

The second pattern was exhibited by EherOBP2, EherOBP4, CubiOBP2, CubiOBP4, and the EherOBP3/CubiOBP3 pair, which had uncertain phylogenetic position, probably because there were no similar OBPs among the Hemiptera in the database. EherOBP2 was distantly related to two mirids and delphacids (bootstrap support 45). CubiOBP2 was distantly related to other hemipteran OBPs (bootstrap support 23). EherOBP4 and CubiOBP4 may be related to three reduviid OBPs (bootstrap support 50 and 69, respectively). The EherOBP3/CubiOBP3 pair mentioned above also had uncertain phylogenetic position (bootstrap support 0). Database enrichment will clarify the phylogenetic relationships of these OBPs.

These results support the hypothesis that within the Heteroptera there has been extensive duplication and differentiation of OBPs [50] especially compared to the conservative evolution in the aphids [69] and Lepidoptera [43]. We found no intraspecific orthologues in the stink bugs, unlike the mirid studies [44,53,71], but a slightly higher rate of orthologues between related species. The absence of intraspecific orthologues might be related to the comparatively lower number of full-length OBPs characterized in the three stink bugs species studied. Additionally, as indicated by the alignment and phylogenetic analyses, the stink bug OBPs are probably not splice variants, which according to Hull et al. [53] can generate intraspecific orthologous OBP clades.

**Fig 8:**
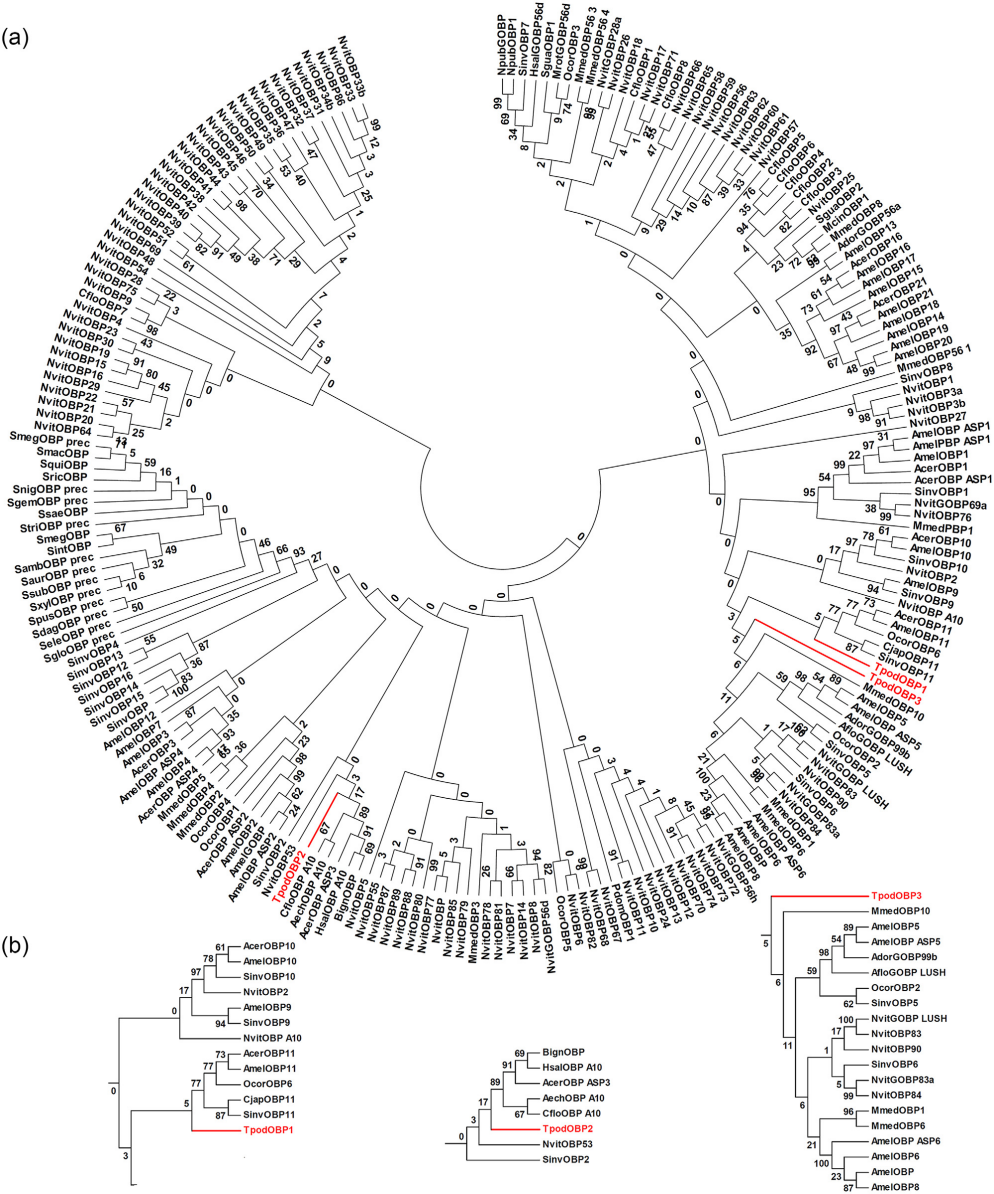
Phylogenetic relationships of: (a) target parasitoid putative OBPs (in red) and 215 putative other hymenopteran OBPs; (b) Detailed relationships of the putative TpodOBPs. The trees were constructed with MEGA 6.06 using a LG+G+I model. Values indicated at the nodes are bootstrap values based on 1,000 replicates presented with 50% cut-off bootstrap value for (a) and no cut-off for (b). The species names are abbreviated with four letters, and their full names with all accession numbers of the OBP amino acid sequences are provided in Table I in S2 File.

The phylogenetic relation of the target parasitoid putative OBPs was made with 215 hymenopteran putative OBPs from 36 species, with a rich representation of *Solenopsis* spp. (18 species), *N*. *vitripennis* putative OBPs (90 units), and *Apis* spp. putative OBPs (42 units) (Fig 8). The OBP phylogeny did not correspond to the known hymenopteran phylogeny. The three TpodOBPs mapped to different parts of the OBP phylogeny and probably are products of different genes for recognition of different odors. None of them are closely related to any of the other hymenopteran OBPs (bootstrap support < 17), and all diverged early from their closest sister OBP. TpodOBP1 and TpodOBP3 were related to OBPs in different hymenopteran families, but with low (5) bootstrap support. TpodOBP2 is most closely related to five other OBPs with low (17) bootstrap support. The phylogenetic pattern for the *T*. *podisi* OBPs differs from other hymenopteran OBPs, which generally have more intra- and interspecific orthologues [50,77], suggesting that evolutionary history of the *T*. *podisi* OBP genes may have been independent of the other identified hymenopteran OBPs.

The high similarities in the primary and tertiary predicted structures indicate that the pairs of OBPs EherOBP3 & CubiOBP3, EherOBP6 & CubiOBP1, TpodOBP1 & EherOBP2, and TpodOBP2 & EherOBP1 might recognize similar odors. The phylogenetic analyses support these inferences. The monophyly between EherOBP3 and EherOBP6 with CubiOBP3 and CubiOBP1, respectively, may imply that they detect similar odors, and that these are different from the odors recognized by the other hemipteran OBPs presently in GenBank. The corresponding parasitoid OBPs, TpodOBP2 and TpodOBP1, have low relatedness to other hymenopteran OBPs, suggesting that they have evolved independent of phylogenetic constraint, and therefore may have evolved to capture host semiochemical cues.

In 2009, Qiao et al. [79] demonstrated that different aphid species shared some highly conserved OBPs, for instance OBP3, which has affinity to sequisterpene (*E*)-β-farnesene (EβF), an aphid alarm pheromone that is released by most aphid species. In addition, lepidopteran PBPs are also similar and monophyletic [43] supporting a hypothesis that OBPs with shared semiochemical ligands are conserved through evolution [54]. Steinbrecht [80] tested the reactivity of an antiserum against the PBP of *Antheraea polyphemus* in pheromone-sensitive sensilla tricodea in nine moth species belonging to six families and three superfamilies of Lepidoptera and concluded that the observed cross-reactivity was related to the chemical relatedness of the pheromones used by the tested species rather than their taxonomic relatedness. The similar OBPs in the two closely related stink bug species, which have broadly overlapping host plant ranges and have similar ecological niches, could suggest that these species can recognize a pheromone component of the other species, such as defense compounds [7,81], to avoid resource competition with the other species, or use it as a cue to locate host plants for mating, feeding and laying eggs. Borges et al. [21,22,82] have shown in field studies that *E*. *heros* sex pheromone baited traps captured not only *E*. *heros*, but also other pentatomids, such as *Edessa meditabunda* and *Piezodorus guildinii*, and platygastrid egg parasitoids. The shared OBP would not be species-specific but rather ligand-specific, which would imply that species sharing a similar ecological niche (such as host plants, natural enemies, defence compounds), could have a similar OBP, no matter how those species are phylogenetically related.

The similar OBPs in the parasitoid and its host could suggest that the parasitoid can recognize a host pheromone, or some other scent that the stink bug uses in its chemical communication. Laboratory bioassays and field tests showed that *T*. *podisi* uses the sex pheromone of *E*. *heros* to locate its hosts [21,22,82]. Therefore, TpodOBP1 and TpodOBP2 might be involved in recognizing a pheromone of *E*. *heros*. Vandermoten et al. [83] found an OBP in the aphid *Sitobion avenae* (SaveOBP3), and two of its predators, the syrphid fly *Episyrphus balteatus* (EbalOBP3) and the ladybird beetle *Harmonia axyridis* (HaxyOBP3), with amino acid sequence similarities ≥ 94%. Both SaveOBP3 and EbalOBP3 recognized a component of the aphid alarm pheromone EβF, which was acting as a kairomone for the natural enemies.

The high primary and tertiary structure similarities between the two pairs of similar OBPs in the stink bugs *E*. *heros* and *C*. *ubica*, may indicate that they have derived from a common ancestor, retaining the same biological function to bind to a ligand perceived or produced in both species. In the case of the parasitoid and host, the parasitoid OBPs may have evolved to become similar to the host OBPs to recognize host semiochemicals. Hence the parasitoid may be eavesdropping on stink bug chemical communication to find suitable hosts to parasitize. Such convergent evolution of a parasitoid OBP would be expected in the tertiary structure, specifically in the OBP domain. However, the OBP domain in insects comprises a uniquely large portion of the protein, so convergent evolution must also act on the OBP primary structure. Overall, these results reinforce that OBP similarity could be more related to specificity to a ligand than to species relatedness. Highly similar OBPs among species might occur via multiple evolutionary processes and provide one pathway to a refined and selective interspecific semiochemical signaling via allomones, kairomones and synomones.

## Conclusion

The transcriptomes of *E*. *heros*, *C*. *ubica* and *D*. *melacanthus* stink bugs were similar to that of other hemipteran species and the transcriptome of *T*. *podisi* parasitoid was similar to other hymenopteran species. Overall, the biological processes and molecular functions were similar among the four species. The most common biological process was related to oxidation-reduction, and the most common molecular function was related to ATP-binding, which have been commonly reported for other insect transcriptomes. Transcriptome analyses enabled the mining of putative OBPs, with a similar number identified from *E*. *heros* and *C*. *ubica* stink bugs, which was higher than from other Hemiptera in the Miridae, Aphididae and Delphacidae families. The lone exception was the mirid *L*. *lineolaris*. Fewer OBPs were identified from *D*. *melacanthus* compared to the other stink bugs, and fewer were from their parasitoid compared to most other Hymenoptera. The full-length putative OBPs had physicochemical properties expected of OBPs, except for an unusual basic pI for one *T*. *podisi* OBP. They largely exhibited the classical Cys-motif pattern, except one minus-C in *D*. *melacanthus* and one plus-C in *E*. *heros* and *C*. *ubica*. High similarity (> 75%) in the 1D and 3D predicted structures were found between two pairs of *E*. *heros* and *C*. *ubica* OBPs, and also between two pairs of *E*. *heros* and its egg parasitoid OBPs, for which the latter had much lower similarity (< 28%) to other hymenopteran OBPs. The 1D and 3D alignments and phylogenetic analyses suggest that these highly similar OBPs might bind to similar semiochemicals. For species sharing a similar ecological niche, such as the stink bugs, this might function for finding shared host plants or recognizing alarm pheromones to shared threats (e.g., natural enemies). For the parasitoid, this might function for recognizing host semiochemical cues to help find hosts.

## Supporting Information

S1 File BLASTx top 5 hits. (XLSX)

S2 File Containing Tables A to I and Figure A. (DOCX)
