# Supplementary material for: Transcriptome-Based Identification of Highly Similar Odorant-Binding Proteins among Neotropical Stink Bugs and Their Egg Parasitoid
Source: PLoS One. 2015 Jul 10;10(7):e0132286. doi: 10.1371/journal.pone.0132286 (PMC4498631; doi:10.1371/journal.pone.0132286)
Supplement: S2 File — (DOCX) [file pone.0132286.s002.docx]

**Supporting Information**

**Transcriptome-based identification of highly similar odorant-binding proteins among Neotropical stink bugs and their egg parasitoid**

Short title: Identification of similar OBPs

Luciana R. Farias^1,2^, Pedro H. C. Schimmelpfeng^1,2^, Roberto C. Togawa^2^, Marcos M. C. Costa^2^, Priscila Grynberg^2^, Natália F. Martins^2^, Miguel Borges^2^, Maria Carolina B. Moraes^2^, Raul A. Laumann^2^, Sônia N. Báo^1^, Débora P. Paula^2^*

*^1^ University of Brasília, Campus Universitário Darcy Ribeiro, Brasília-DF, 70910-900, Brazil, and ^2^ Embrapa Genetic Resources and Biotechnology, Parque Estação Biológica, W5 Norte, P.O. Box 02372, Brasília, DF, 70770-917, Brazil*

* [debora.pires@embrapa.br](mailto:debora.pires@embrapa.br)

Phone: +55 (61) 34484929; Fax: +55 (61) 34484672

**Table A. I-Tasser estimated indexes of the quality of the predicted models.**

| **Protein** | **C-Score** | **Expected TM score** | **Expected RMSD** | **Template PDB codes** |
| --- | --- | --- | --- | --- |
| Eher OBP3 | 0.76 | 0.82+-0.09 | 2.6+-1.9 | 3V2L |
| Eher OBP4 | 0.66 | 0.8 +- 0.09 | 2.6+-2.0 | 3V2L |
| Eher OBP5 | -2.09 | 0.47+-0.15 | 9.7+-4.6 | 3V2L |
| Eher OBP6 | -1.29 | 0.55+-0.15 | 8.3+-4.5 | 4IJ7A |
| Cubi OBP1 | -1,26 | 0.56+-0.15 | 8.2+-4.5 | 4IJ7A |
| Cubi OBP2 | -0.98 | 0.59+-0.14 | 6.7+-4.0 | 3V2L |
| Cubi OBP3 | -0.79 | 0.61+-0.14 | 6.3+-3.8 | 3R72A |
| Cubi OBP4 | -0.78 | 0.61+-0.14 | 6.4+-3.9 | 3R72A |
| Dimel OBP1 | -0.46 | 0.65+-0.13 | 5.6+-3.5 | 3R72A |
| Tpod OBP1 | -0.58 | 0.64+-0.13 | 5.7+-3.6 | 3D74A |
| Tpod OBP2 | -0.83 | 0.61+-0.14 | 6.5+-3.9 | 3R72A |

C-score is a confidence score for estimating the quality of predicted models by I-TASSER. It is calculated based on the significance of threading template alignments and the convergence parameters of the structure assembly simulations. C-score is typically in the range of -5 and 2, where a C-score of higher value signifies a model with a high confidence.

TM-score is a recently proposed scale for measuring the structural similarity between two structures (see Zhang and Skolnick 2004). The purpose of proposing TM-score is to solve the problem of root mean squared deviation (RMSD), which is sensitive to the local error. Because RMSD is an average deviation of all residue pairs in two structures, a local error (e.g. a misorientation of the tail) will create a large RMSD value although the global topology is correct. In the TM-score, however, small deviations are weighted more heavily than large deviations, which make the score insensitive to local modeling error. A TM-score >0.5 indicates a model of correct topology and a TM-score<0.17 means random similarity. These cutoffs do not depend on the protein length.

I-TASSER generates full-length models of proteins by excising continuous fragments from threading alignments and then reassembling them using replica-exchanged Monte Carlo simulations. Low temperature replicas (decoys) generated during the simulation are clustered by SPICKER and the top five cluster centroids are selected for generating full atomic models. The cluster density is defined as the number of structure decoys in a unit of space in the SPICKER cluster. A higher cluster density means the structure occurs more often in the simulation trajectory and therefore signifies a better quality model. The values in the second-last column of the above mentioned table represents the structural RMSD between the target and the template. The last column represents the template PDB codes (Zhang 2008; Roy et al. 2010, 2012).

**References**

Roy A, Kucukural A, Zhang Y (2010) I-TASSER: a unified platform for automated protein structure and function prediction. Nat. Protoc. 5: 725-738.

Roy A, Yang J, Zhang Y (2012) COFACTOR: an accurate comparative algorithm for structure-based protein function annotation. Nucleic Acids Res. 40: W471-W477.

Zhang Y (2008) I-TASSER server for protein 3D structure prediction. BMC Bioinformatics 9: 40-48.

Zhang Y, Skolnick J (2004) Scoring function for automated assessment of protein structure template quality. Proteins 57: 702-710.

**Table B. Number of transcripts (and percent) for BLAST, mapping and annotation analyses of the transcriptome for each species.**

|  | ***E. heros*** | ***D. melacanthus*** | ***C. ubica*** | ***T. podisi*** |
| --- | --- | --- | --- | --- |
| Without BLAST hits | 8,662 (43.98%) | 4,058 (45.87%) | 26,991 (64.48%) | 19,605 (38.27%) |
| With BLAST results | 5,142 (26.11%) | 2,346 (26.52%) | 7,370 (17.61%) | 22,926 (44.76%) |
| With Mapping results | 2,081 (10.57%) | 763 (8.62%) | 2,905 (6.94%) | 3,153 (6.16%) |
| Annotated sequences | 3,812 (19.35%) | 1,680 (18.99%) | 4,594 (10.97%) | 5,540 (10.82%) |
| Total sequences | 19,697 | 8,847 | 41,860 | 51,224 |

**Table C. Number (and % completeness) of CEGs (Core Eukaryotic Genes) mapped to the contigs of the stink bugs *E. heros*, *C. ubica*, *D. melacanthus* and the parasitoid *T. podisi*.** Group 1 is represented by the least conserved CEGs, whereas group 4 has the most conserved ones. #Prots= number of 248 ultra-conserved CEGs present in the transcriptome; % compl.= percentage of 248 ultra-conserved CEGs present; Total= total number of CEGs present including putative orthologs; Average= average number of orthologs per CEG; % Ortho= percentage of detected CEGs that have more than 1 ortholog.

| **Group** | ***E. heros*** | | | ***C. ubica*** | | | ***D. melacanthus*** | | | ***T. podisi*** | | |
| --- | --- | --- | --- | --- | --- | --- | --- | --- | --- | --- | --- | --- |
|  | **#Prots**  **(% compl.)** | **# Total** | **Average**  **(% Ortho)** | **#Prots**  **(% compl.)** | **# Total** | **Average**  **(% Ortho)** | **#Prots**  **(% compl.)** | **# Total** | **Average**  **(% Ortho)** | **#Prots**  **(% compl.)** | **# Total** | **Average**  **(% Ortho)** |
| 1 | 22 (33.33) | 52 | 2.36 (68.18) | 24 (36.36) | 58 | 2.42 (75.00) | 7 (10.61) | 13 | 1.86 (57.14) | 58 (87.88) | 183 | 3.16 (79.31) |
| 2 | 16 (28.57) | 48 | 3.00 (75.00) | 24 (42.86) | 59 | 2.46 (62.50) | 3 (5.36) | 4 | 1.33 (33.33) | 52 (92.86) | 169 | 3.25 (82.69) |
| 3 | 31 (50.82) | 77 | 2.48 (70.97) | 32 (52.46) | 84 | 2.62 (71.88) | 12 (19.67) | 33 | 2.75 (75.00) | 55 (90.16) | 210 | 3.82 (89.09) |
| 4 | 41 (63.08) | 88 | 2.15 (53.66) | 44 (67.69) | 113 | 2.57 (65.91) | 15 (23.08) | 37 | 2.47 (73.33) | 58 (89.23) | 220 | 3.79 (91.38) |
| Total | 110 (44.35) |  |  | 124 (50.00) |  |  | 37 (14.92) |  |  | 223 (89.92) |  |  |

**Table D. Contigs from the antennae of male and female 12 day-old virgin adults of the stink bug *E. heros* with similarity to olfaction related genes.** The contigs that contain the full-length OBPs are represented with the accession number in bold.

| **Accession code** | **Similarity** | **Length (bp)** | **Hits** | **Min. E-value** | **Mean similarity (%)** |
| --- | --- | --- | --- | --- | --- |
| GBER01005583 | CSP | 629 | 10 | 4.1E-24 | 70.4 |
| GBER01005618 | CSP | 536 | 10 | 1.9E-25 | 77.1 |
| GBER01012184 | CSP | 407 | 1 | 6.3E-9 | 59.0 |
| GBER01018566 | CSP | 399 | 10 | 9.8E-34 | 81.0 |
| GBER01001032 | CSP1 | 690 | 10 | 2.6E-34 | 75.1 |
| GBER01011255 | CSP 1 and 3 | 574 | 10 | 1.5E-17 | 80.3 |
| GBER01009771 | CSP1 and 2 | 447 | 10 | 5.4E-24 | 65.1 |
| GBER01010357 | CSP2 | 319 | 10 | 1.7E-17 | 66.0 |
| GBER01010544 | CSP2 | 527 | 10 | 2.2E-15 | 62.8 |
| GBER01014200 | CSP2 | 443 | 8 | 2.9E-25 | 70.0 |
| GBER01016775 | CSP2 | 487 | 10 | 4.9E-33 | 80.8 |
| GBER01017174 | CSP2 | 334 | 10 | 9.6E-34 | 75.7 |
| GBER01000567 | OBP | 726 | 9 | 1.8E-15 | 53.4 |
| GBER01001615 | OBP | 557 | 1 | 2.3E-11 | 55.0 |
| GBER01001803 | OBP | 828 | 6 | 1.0E-15 | 48.8 |
| GBER01003358 | OBP | 373 | 1 | 1.8E-11 | 57.0 |
| GBER01005551 | OBP | 718 | 6 | 3.5E-14 | 58.3 |
| GBER01006153 | OBP | 859 | 10 | 3.3E-20 | 51.6 |
| GBER01006190 | OBP | 490 | 10 | 1.6E-28 | 62.4 |
| GBER01007924 | OBP | 1039 | 10 | 1.4E-49 | 69.1 |
| GBER01014089 | OBP | 338 | 3 | 6.7E-27 | 78.3 |
| **GBER01016586** | OBP | 617 | 10 | 2.1E-44 | 67.0 |
| GBER01018275 | OBP | 425 | 10 | 1.6-15 | 52.5 |
| GBER01000050 | OBP1 | 476 | 2 | 4.8E-9 | 63.5 |
| GBER01017348 | OBP1 | 387 | 3 | 6.7E-27 | 78.3 |
| GBER01004408 | OBP2 | 542 | 3 | 6.1E-26 | 71.7 |
| GBER01010977 | OBP2 | 384 | 4 | 2.1E-35 | 79.6 |
| GBER01011319 | OBP2 and 4 | 341 | 3 | 3.2E-13 | 82.0 |
| GBER01008196 | PBP3 and OBP5 | 726 | 10 | 1.7E-32 | 70.0 |
| **GBER01008711** | OBP3 | 652 | 8 | 1.2E-16 | 52.6 |
| GBER01010155 | OBP3 | 532 | 1 | 9.2E-9 | 48.0 |
| GBER01003441 | OBP5 | 351 | 7 | 5.4E-14 | 50.7 |
| **GBER01006133** | OBP6 | 514 | 8 | 5.2E-11 | 50.2 |
| **GBER01013326** | OBP7 | 736 | 1 | 2.3E-26 | 52.0 |
| GBER01006979 | OR1-like | 1096 | 10 | 4.3E-16 | 48.9 |
| GBER01018823 | OR10 | 341 | 1 | 4.8E-9 | 62.0 |
| GBER01018766 | OR43a | 650 | 10 | 5.4E-12 | 46.5 |
| GBER01000496 | OR47 | 1191 | 10 | 7.9E-14 | 41.9 |
| GBER01017355 | OR85b | 845 | 10 | 2.6E-14 | 43.6 |

**Table E. Contigs from the antennae of male and female 12 day-old virgin adults of the stink bug *C. ubica* with similarity to olfaction related genes.** The contigs that contain the full-length OBPs are represented with the accession number in bold.

| **Accession code** | **Similarity** | **Length (bp)** | | **Hits** | | **Min. E-value** | | **Mean similarity (%)** | |
| --- | --- | --- | --- | --- | --- | --- | --- | --- | --- |
| GBFA01009672 | CSP | 510 | 10 | | 1.7E-38 | | 75.0 | |  |
| GBFA01015066 | CSP | 396 | 10 | | 6.5E-22 | | 68.5 | |  |
| GBFA01011367 | CSP1 | 481 | 5 | | 7.3E-12 | | 69.2 | |  |
| GBFA01030507 | CSP1 | 395 | 10 | | 4.9E-25 | | 82.1 | |  |
| GBFA01013124 | CSP1 | 537 | 10 | | 1.7E-29 | | 76.0 | |  |
| GBFA01015075 | CSP1 | 442 | 10 | | 6.6E-30 | | 76.3 | |  |
| GBFA01015168 | CSP1 | 432 | 10 | | 3.3E-37 | | 78.7 | |  |
| GBFA01033041 | CSP1 | 387 | 10 | | 4.2E-29 | | 81.7 | |  |
| GBFA01029042 | CSP2 | 375 | 10 | | 3.4E-42 | | 75.5 | |  |
| GBFA01038670 | CSP2 | 501 | 10 | | 4.6E-20 | | 55.3 | |  |
| GBFA01041041 | CSP2 | 507 | 10 | | 6.1E-94 | | 73.8 | |  |
| GBFA01018326 | CSP3 | 340 | 10 | | 6.6E-35 | | 77.6 | |  |
| GBFA01035760 | CSP4 | 840 | 10 | | 7.3E-17 | | 64.6 | |  |
| GBFA01009611 | CSP11 | 763 | 10 | | 1.4E-29 | | 76.8 | |  |
| GBFA01026402 | CSP receptor | 656 | 10 | | 3.2E-12 | | 98.2 | |  |
| GBFA01004332 | OBP | 1257 | 5 | | 7.4E-10 | | 58.4 | |  |
| GBFA01004415 | OBP | 717 | 3 | | 3.8E-31 | | 73.0 | |  |
| GBFA01008431 | OBP | 615 | 4 | | 6.6E-20 | | 60.8 | |  |
| GBFA01013912 | OBP | 390 | 3 | | 9.7E-10 | | 58.0 | |  |
| GBFA01014515 | OBP | 306 | 5 | | 4.1E-16 | | 65.8 | |  |
| GBFA01015561 | OBP | 582 | 7 | | 1.3E-13 | | 62.3 | |  |
| **GBFA01018695** | OBP | 650 | 7 | | 1.8E-12 | | 50.1 | |  |
| GBFA01019136 | OBP | 364 | 1 | | 4.6E-20 | | 71.0 | |  |
| GBFA01021331 | OBP2 | 846 | 10 | | 4.8E-40 | | 59.6 | |  |
| GBFA01022585 | OBP2 | 417 | 1 | | 1.3E-9 | | 52.0 | |  |
| GBFA01020472 | OBP | 563 | 10 | | 2.5E-17 | | 65.3 | |  |
| GBFA01031739 | OBP | 857 | 10 | | 3.5E-46 | | 67.3 | |  |
| GBFA01032703 | OBP | 777 | 10 | | 4.2E-13 | | 50.8 | |  |
| GBFA01033237 | OBP | 714 | 1 | | 6.3E-11 | | 68.0 | |  |
| GBFA01034469 | OBP | 820 | 1 | | 1.5E-11 | | 65.0 | |  |
| GBFA01025424 | OBP1 | 443 | 2 | | 1.7E-9 | | 59.0 | |  |
| GBFA01004357 | OBP2 | 479 | 2 | | 3.3E-10 | | 56.0 | |  |
| **GBFA01032915** | OBP2 | 560 | 4 | | 6.3E-17 | | 55.2 | |  |
| GBFA01015182 | OBP2 | 521 | 1 | | 2.1E-10 | | 66.0 | |  |
| GBFA01020615 | OBP3 | 441 | 5 | | 4.2E-13 | | 54.4 | |  |
| GBFA01024842 | OBP2 | 583 | 3 | | 1.0E-17 | | 80.7 | |  |
| GBFA01035284 | OBP3 | 532 | 1 | | 4.3E-9 | | 56.0 | |  |
| **GBFA01016877** | OBP6 | 928 | 2 | | 7.1E-11 | | 52.5 | |  |
| **GBFA01004373** | OBP7 | 877 | 1 | | 3.8E-27 | | 53.0 | |  |
| GBFA01004328 | OBP14 | 786 | 5 | | 4.5E-10 | | 57.0 | |  |
| GBFA01029530 | OR | 351 | 2 | | 2.2E-9 | | 55.0 | |  |
| GBFA01010571 | OR | 993 | 10 | | 3.5E-14 | | 48.2 | |  |
| GBFA01035378 | OR | 521 | 10 | | 1.5E-13 | | 45.1 | |  |
| GBFA01006113 | OR1-like | 1207 | 2 | | 1.1E-10 | | 58.5 | |  |
| GBFA01025843 | OR2a-like | 503 | 9 | | 6.7E-11 | | 47.8 | |  |
| GBFA01010627 | OR17 | 711 | 10 | | 3.3E-11 | | 51.8 | |  |
| GBFA01025450 | OR25 | 609 | 3 | | 1.7E-9 | | 51.7 | |  |
| GBFA01037414 | OR38 | 426 | 10 | | 8.1E-12 | | 60.8 | |  |
| GBFA01040723 | OR82 | 498 | 10 | | 3.8E-14 | | 52.7 | |  |
| GBFA01002375 | OR92a | 566 | 5 | | 1.7E-9 | | 51.0 | |  |

**Table F. Contigs from the antennae of male and female 12 day-old virgin adults of the stink bug *D. melacanthus* with similarity to olfaction related genes.** The contigs that contain the full-length OBPs are represented with the accession number in bold.

| **Accession code** | | **Similarity** | | **Length (bp)** | | **Hits** | | **Min. E-value** | | **Mean similarity (%)** | |
| --- | --- | --- | --- | --- | --- | --- | --- | --- | --- | --- | --- |
| GBES01005338 | CSP | | 331 | | 10 | | 4.7E-33 | | 85.9 | |  |
| GBES01006562 | CSP | | 564 | | 10 | | 2.0E-10 | | 57.0 | |  |
| GBES01000905 | CSP1 | | 440 | | 10 | | 1.1E-40 | | 79.4 | |  |
| GBES01007090 | CSP | | 311 | | 10 | | 3.0E-19 | | 74.8 | |  |
| GBES01000993 | CSP2 | | 325 | | 10 | | 2.0E-10 | | 62.3 | |  |
| GBES01001189 | CSP2 | | 333 | | 10 | | 1.9E-13 | | 65.0 | |  |
| GBES01007172 | CSP2 | | 326 | | 10 | | 3.5E-23 | | 78.6 | |  |
| GBES01000338 | OBP | | 441 | | 8 | | 2.9E-14 | | 56.8 | |  |
| GBES01000353 | OBP | | 313 | | 1 | | 5.0E-18 | | 80.0 | |  |
| GBES01000912 | OBP | | 351 | | 6 | | 2.4E-16 | | 67.5 | |  |
| GBES01000970 | OBP | | 492 | | 4 | | 3.5E-31 | | 70.0 | |  |
| GBES01002143 | OBP | | 469 | | 10 | | 9.4E-29 | | 63.9 | |  |
| GBES01002865 | OBP | | 556 | | 10 | | 2.4E-32 | | 76.0 | |  |
| GBES01004331 | OBP | | 667 | | 2 | | 5.0E-16 | | 61.5 | |  |
| GBES01007180 | OBP | | 460 | | 4 | | 4.4E-10 | | 60.2 | |  |
| **GBES01006465** | OBP2 | | 441 | | 10 | | 2.7E-41 | | 60.1 | |  |

**Table G. Contigs from the whole body of 20 day-old female of the parasitoid *T. podisi* with similarity to olfaction related genes.** The contigs that contain the full-length OBPs are represented with the accession number in bold.

| **Accession code** | | **Similarity** | | **Length (bp)** | | **Hits** | | **Min. E-value** | | **Mean similarity (%)** | |
| --- | --- | --- | --- | --- | --- | --- | --- | --- | --- | --- | --- |
| GBEU01002338 | CSP | | 1039 | | 10 | | 1.6E-17 | | 71.1 | |  |
| GBEU01000146 | CSP1 | | 425 | | 1 | | 6.4E-9 | | 75.0 | |  |
| GBEU01044678 | CSP1 | | 301 | | 10 | | 2.5E-10 | | 79.9 | |  |
| GBEU01046987 | CSP1 | | 416 | | 2 | | 4.0E-16 | | 62.0 | |  |
| GBEU01043137 | CSP2 | | 392 | | 10 | | 2.5E-10 | | 53.5 | |  |
| **GBEU01036727** | OBP | | 472 | | 5 | | 1.7E-62 | | 66.8 | |  |
| GBEU01002747 | PBP1 | | 727 | | 10 | | 6.7E-15 | | 67.8 | |  |
| GBEU01004571 | PBP3 | | 522 | | 10 | | 6.1E-20 | | 66.1 | |  |
| GBEU01026442 | PBP3 | | 496 | | 10 | | 1.3E-14 | | 49.6 | |  |
| **GBEU01046281** | PBP3 | | 606 | | 7 | | 1.0E-11 | | 51.6 | |  |
| **GBEU01005726** | OBP4 | | 541 | | 10 | | 4.2E-44 | | 66.6 | |  |
| GBEU01035794 | OBPa10 | | 781 | | 10 | | 2.9E-38 | | 79.9 | |  |
| GBEU01035690 | OR2-like | | 618 | | 2 | | 4.9E-9 | | 53.0 | |  |
| GBEU01036816 | OR13a | | 365 | | 2 | | 3.2E-16 | | 81.0 | |  |
| GBEU0104899 | OR13a-like | | 395 | | 10 | | 3.9E-14 | | 62.8 | |  |
| GBEU01050959 | OR63a-like | | 352 | | 10 | | 9.2E-16 | | 59.7 | |  |
| GBEU01046354 | OR257 | | 399 | | 10 | | 1.1E-11 | | 58.3 | |  |
| GBEU01049919 | OR289 | | 314 | | 3 | | 6.9E-24 | | 65.3 | |  |
| GBEU01050340 | OR289 | | 349 | | 3 | | 2.3E-14 | | 56.3 | |  |

**Table H.** **Identity matrix (%) for all the orthologous sequences used in the alignments with the target stink bugs (*E. heros*, *C. ubica* and *D. melacanthus*) and parasitoid (*T. podisi*) putative OBPs.**

**Table I. Accession numbers of the OBP amino acid sequences used in this work.**

| **Species** | **OBP name abbreviation** | **GenBank accession code** |
| --- | --- | --- |
| *Apis cerana* | AcerOBP_ASP1 | ABD97847.1 |
|  | AcerOBP_ASP2 | ABD97844.1 |
|  | AcerOBP_ASP3 | ABD97846.2 |
|  | AcerOBP_ASP4 | AAR83081.1 |
|  | AcerOBP1 | AEZ65022.1 |
|  | AcerOBP3 | AHN15444.1 |
|  | AcerOBP10 | AEY59710.1 |
|  | AcerOBP11 | AGQ03796.1 |
|  | AcerOBP16 | AEY59889.1 |
|  | AcerOBP21 | AEY61867.1 |
| *Anomala corpulenta* | AcorPBP | AGZ93682.1 |
| *Aphis craccivora* | AcraOBP2 | CAR85658.1 |
| *Apis dorsata* | AdorGOBP56a | XP_006609096.1 |
|  | AdorGOBP99b | XP_006616939.1 |
| *Acromyrmex echinatior* | AechOBP_A10 | EGI64540.1 |
| *Aphis fabae* | AfabOBP2 | CAR85656.1 |
|  | AfabOBP8 | CAR85657.1 |
| *Apis florea* | AfloGOBP_LUSH | XP_003690412.1 |
| *Aphis glycines* | AglyOBP2 | AHJ80888.1 |
|  | AglyOBP3 | AHJ80889.1 |
|  | AglyOBP4 | AHJ80890.1 |
|  | AglyOBP5 | AHJ80891.1 |
|  | AglyOBP6 | AHJ80892.1 |
|  | AglyOBP7 | AHJ80893.1 |
|  | AglyOBP8 | AHJ80894.1 |
|  | AglyOBP9 | AHJ80895.1 |
|  | AglyOBP10 | AHJ80896.1 |
|  | AglyOBP11 | AHJ80897.1 |
| *Aphis gossypii* | AgosOBP | ACI30678.1 |
|  | AgosOBP BP2 | AGP04985.1 |
|  | AgosOBP BP3 | AGP04977.1 |
|  | AgosOBP BP4 | AGP04978.1 |
|  | AgosOBP BP5 | AGP04983.1 |
|  | AgosOBP BP6 | AGP04979.1 |
|  | AgosOBP BP7 | AGP04980.1 |
|  | AgosOBP BP8 | AGP04981.1 |
|  | AgosOBP BP9 | AGP04984.2 |
|  | AgosOBP BP10 | AGP04982.1 |
| *Adelphocoris lineolatus* | AlinOBP | ACT83085.1 |
|  | AlinOBP1 | ACT83086.1 |
|  | AlinOBP2 | ACZ58028.1 |
|  | AlinOBP3 | ACZ58029.1 |
|  | AlinOBP4 | ACZ58030.1 |
|  | AlinOBP5 | ACZ58031.1 |
|  | AlinOBP6 | ACZ58032.1 |
|  | AlinOBP7 | ACZ58085.1 |
|  | AlinOBP8 | ACZ58079.1\| |
|  | AlinOBP9 | ACZ58080.1 |
|  | AlinOBP10 | ACZ58081.1 |
|  | AlinOBP11 | ACZ58082.1 |
|  | AlinOBP12 | ACZ58083.1 |
|  | AlinOBP13 | ACZ58084.1 |
|  | AlinOBP14 | ACZ58086.1 |
|  | AlinOBP15 | ACZ58033.1 |
|  | AlinOBP16 | ACZ58034.1 |
| *Apolygus lucorum* | AlucOBP1 | AEA07705.1 |
|  | AlucOBP2 | AEA07706.1 |
|  | AlucOBP3 | AEA07661.1 |
|  | AlucOBP4 | AEP95761.1 |
|  | AlucOBP5 | AEP95759.1 |
|  | AlucOBP6 | AEA07664.1 |
|  | AlucOBP7 | AFJ54048.1 |
|  | AlucOBP8 | AFJ54049.1 |
|  | AlucOBP9 | AFJ54050.1 |
|  | AlucOBP10 | AFJ54051.1 |
|  | AlucOBP11 | AFJ54052.1 |
|  | AlucOBP12 | AFJ54053.1 |
| *Apis mellifera* | AmelGOBP | 1TUJ\|A |
|  | AmelOBP | AF339140_1 |
|  | AmelOBP_ASP1 | AF393494_1 |
|  | AmelOBP_ASP2 | AF393493_1 |
|  | AmelOBP_ASP4 | AF393492_1 |
|  | AmelOBP_ASP5 | AF393497_1 |
|  | AmelOBP_ASP6 | AF393496_1 |
|  | AmelOBP1 | NP_001011590.1 |
|  | AmelOBP2 | NP_001011591.1 |
|  | AmelOBP3 | ABD92639.1 |
|  | AmelOBP4 | NP_001011589.1 |
|  | AmelOBP5 | NP_001011588.1 |
|  | AmelOBP6 | XP_006567383.1 |
|  | AmelOBP7 | NP_001035310.1 |
|  | AmelOBP8 | NP_001164515.1 |
|  | AmelOBP9 | ABD92641.1 |
|  | AmelOBP10 | ABD92642.1 |
|  | AmelOBP11 | NP_001035316.1 |
|  | AmelOBP12 | ABD92644.1 |
|  | AmelOBP13 | ABD92645.1 |
|  | AmelOBP14 | ABD92646.1 |
|  | AmelOBP15 | ABD92647.1 |
|  | AmelOBP16 | ABD92648.1 |
|  | AmelOBP17 | ABD92649.1 |
|  | AmelOBP18 | ABD92650.1 |
|  | AmelOBP19 | ABD92651.1 |
|  | AmelOBP20 | ABD92652.1 |
|  | AmelOBP21 | ABD92653.1 |
|  | AmelPBP_ASP1 | AAD51944.1 |
| *Anomala osakana* | AosaPBP | AAC63437.1 |
| *Acyrthosiphon pisum* | ApisGOBP69a | XP 008182417.1 |
|  | ApisOBP | ACI30694.1 |
|  | ApisOBP A10 | XP 001947629.1 |
|  | ApisOBP1 | CAR85628.1 |
|  | ApisOBP2 | CAR85629.1 |
|  | ApisOBP3 | AGE97633.1 |
|  | ApisOBP4 | NP 001153530.1 |
|  | ApisOBP5 | AGE97635.1 |
|  | ApisOBP6 | XP 008181869.1 |
|  | ApisOBP7 | AGE97637.1 |
|  | ApisOBP8 | CAR85635.1 |
|  | ApisOBP9 | NP 001153535.1 |
|  | ApisOBP10 | AGE97640.1 |
|  | ApisOBP11 | CAX63068.1 |
|  | ApisOBP12 | CAX63069.1 |
|  | ApisOBP13 | CAX63070.1 |
| *Aulacorthum solani* | AsolOBP7 | AHH34994.1 |
| *Adelphocoris suturalis* | AsutOBP6 | AHJ81241.1 |
|  | AsutOBP7 | AHJ81239.1 |
|  | AsutOBP8 | AHJ81242.1 |
|  | AsutOBP10 | AHJ81240.1 |
|  | AsutOBP11 | AHJ81243.1 |
|  | AsutOBP12 | AHJ81244.1 |
| *Brevicoryne brassicae* | BbraOBP3 | AEX65667.1 |
| *Bombus ignitus* | BignOBP | ACA64427.1 |
| *Bemisia tabaci* | BtabOBP | AER27564.1 |
| *Camponotus floridanus* | CfloOBP_A10 | EFN64584.1 |
| *Copidosoma floridanum* | CfloOBP1 | ABB58734.1 |
|  | CfloOBP2 | ABB58735.1 |
|  | CfloOBP3 | AHE40945.1 |
|  | CfloOBP4 | AHE40946.1 |
|  | CfloOBP5 | AHE40947.1 |
|  | CfloOBP6 | AHE40948.1 |
|  | CfloOBP7 | AHE40949.1 |
|  | CfloOBP8 | AHE40950.1 |
| *Camponotus japonicus* | CjapOBP11 | BAO48209.1 |
| *Diaphorina citri* | DcitOBP prec | ABG81983.1 |
| *Drepanosiphum platanoidis* | DplaOBP3 | AEX65663.1 |
| *Euschistus heros* | EherOBP1 | ADJ18275.1 |
|  | EherOBP2 | ADO24165.1 |
| *Frankliniella occidentalis* | FoccPBP | AEP27187.1 |
| *Harpegnathos saltator* | HsalGOBP56d | EFN84434.1 |
|  | HsalOBP_A10 | EFN75075.1 |
| *Heptophylla picea* | HpicOBP1 | BAC07270.1 |
| *Lygus lineolaris* | LlinOBP1 | AHF71028.1 |
|  | LlinOBP2 | AHF71029.1 |
|  | LlinOBP3 | AHF71030.1 |
|  | LlinOBP4 | AHF71031.1 |
|  | LlinOBP5 | AHF71032.1 |
|  | LlinOBP6 | AHF71033.1 |
|  | LlinOBP7 | AHF71034.1 |
|  | LlinOBP8 | AHF71035.1 |
|  | LlinOBP9 | AHF71036.1 |
|  | LlinOBP10 | AHF71037.1 |
|  | LlinOBP11 | AHF71038.1 |
|  | LlinOBP12 | AHF71039.1 |
|  | LlinOBP13 | AHF71040.1 |
|  | LlinOBP14 | AHF71041.1 |
|  | LlinOBP15 | AHF71042.1 |
|  | LlinOBP16 | AHF71043.1 |
|  | LlinOBP18 | AHF71046.1 |
|  | LlinOBP19 | AHF71049.1 |
|  | LlinOBP27 | AHF71058.1 |
|  | LlinOBP29 | AHF71060.1 |
|  | LlinOBP30 | AHF71061.1 |
|  | LlinOBP31 | AHF71062.1 |
|  | LlinOBP32 | AHF71063.1 |
|  | LlinOBP33 | AHF71054.1 |
| *Locusta migratoria* | LmigOBP5 | AEX33161.1 |
| *Laodelphax striatella* | LstrOBP1 | AEQ19907.1 |
|  | LstrOBP2 | AEQ19908.1 |
|  | LstrOBP3 | AEQ19909.1 |
|  | LstrOBP4 | AEQ19910.1 |
|  | LstrOBP5 | AEQ19911.1 |
| *Macrocentrus cingulum* | McinOBP1 | AHL25274.1 |
| *Metopolophium dirhodum* | MdirOBP1 | CAR85638.1 |
|  | MdirOBP2 | CAR85639.1 |
|  | MdirOBP3 | CAX63256.1 |
|  | MdirOBP4 | CAR85640.1 |
|  | MdirOBP5 | CAR85641.1 |
|  | MdirOBP6 | CAR85642.1 |
|  | MdirOBP8 | CAR85643.1 |
| *Microplitis mediator* | MmedOBP1 | ABM05968.2 |
|  | MmedOBP10 | AEO27860.1 |
|  | MmedOBP2 | ABM05969.1 |
|  | MmedOBP3 | ABM05970.1 |
|  | MmedOBP4 | ABM05971.2 |
|  | MmedOBP5 | ABM05972.1 |
|  | MmedOBP56_1 | EZA45198.1 |
|  | MmedOBP56_3 | EZA45302.1 |
|  | MmedOBP56_4 | EZA45303.1 |
|  | MmedOBP6 | ABO15559.1 |
|  | MmedOBP8 | AEF14409.1 |
|  | MmedPBP1 | ABM05973.2 |
| *Myzus persicae* | MperOBP | ACI30684.1 |
|  | MperOBP4 | CAR85645.1 |
|  | MperOBP6 | CAR85646.1 |
|  | MperOBP7 | CAR85647.1 |
|  | MperOBP8 | CAR85648.1 |
|  | MperOBP10 | CAR85649.1 |
| *Megachile rotundata* | MrotGOBP56d | XP_003708550.1 |
| *Megoura viciae* | MvicOBP1 | CAR85650.1 |
|  | MvicOBP2 | CAR85651.1 |
|  | MvicOBP5 | CAR85652.1 |
|  | MvicOBP8 | CAR85653.1 |
|  | MvicOBP10 | CAX63260.1 |
| *Nilaparvata lugens* | NlugOBP1 | ACI30679.1 |
|  | NlugOBP2 | ACI30680.1 |
|  | NlugOBP3 | ACI30681.2 |
| *Nylanderia pubens* | NpubGOBP | ADX36426.1 |
|  | NpubOBP1 | ADE27967.1 |
| *Nasonovia ribisnigri* | NribOBP2 | CAR85654.1 |
|  | NribOBP3 | CAX63257.1 |
|  | NribOBP5 | CAX63258.1 |
|  | NribOBP7 | CAX63259.1 |
|  | NribOBP8 | CAR85655.1 |
| *Nasonia vitripennis* | NvitGOBP_LUSH | XP_001603472.2 |
|  | NvitGOBP28a | XP_001601182.1 |
|  | NvitGOBP56d | XP_001600573.1 |
|  | NvitGOBP56h | XP_008207799.1 |
|  | NvitGOBP69a | XP_001600769.1 |
|  | NvitGOBP83a | XP_001603497.1 |
|  | NvitOBP | ADK73604.1 |
|  | NvitOBP_A10 | XP_001600111.2 |
|  | NvitOBP1 | CCD17770.1 |
|  | NvitOBP2 | CCD17771.1 |
|  | NvitOBP4 | CCD17773.1 |
|  | NvitOBP5 | CCD17774.1 |
|  | NvitOBP6 | CCD17775.1 |
|  | NvitOBP7 | CCD17776.1 |
|  | NvitOBP8 | CCD17777.1 |
|  | NvitOBP9 | CCD17778.1 |
|  | NvitOBP10 | CCD17779.1 |
|  | NvitOBP11 | CCD17780.1 |
|  | NvitOBP12 | CCD17781.1 |
|  | NvitOBP13 | CCD17782.1 |
|  | NvitOBP14 | CCD17783.1 |
|  | NvitOBP15 | CCD17784.1 |
|  | NvitOBP16 | CCD17785.1 |
|  | NvitOBP17 | CCD17786.1 |
|  | NvitOBP18 | CCD17787.1 |
|  | NvitOBP19 | CCD17788.1 |
|  | NvitOBP20 | CCD17789.1 |
|  | NvitOBP21 | CCD17790.1 |
|  | NvitOBP22 | CCD17791.1 |
|  | NvitOBP23 | CCD17792.1 |
|  | NvitOBP24 | CCD17793.1 |
|  | NvitOBP25 | CCD17794.1 |
|  | NvitOBP26 | CCD17795.1 |
|  | NvitOBP27 | CCD17796.1 |
|  | NvitOBP28 | CCD17797.1 |
|  | NvitOBP29 | CCD17798.1 |
|  | NvitOBP30 | CCD17799.1 |
|  | NvitOBP31 | CCD17800.1 |
|  | NvitOBP32 | CCD17801.1 |
|  | NvitOBP33a | CCD17802.1 |
|  | NvitOBP33b | CCD17861.1 |
|  | NvitOBP34b | CCD17862.1 |
|  | NvitOBP35 | CCD17804.1 |
|  | NvitOBP36 | CCD17805.1 |
|  | NvitOBP37 | CCD17806.1 |
|  | NvitOBP38 | CCD17807.1 |
|  | NvitOBP39 | CCD17808.1 |
|  | NvitOBP3a | CCD17772.1 |
|  | NvitOBP3b | CCD17860.1 |
|  | NvitOBP40 | CCD17809.1 |
|  | NvitOBP41 | CCD17810.1 |
|  | NvitOBP42 | CCD17811.1 |
|  | NvitOBP43 | CCD17812.1 |
|  | NvitOBP44 | CCD17813.1 |
|  | NvitOBP45 | CCD17814.1 |
|  | NvitOBP46 | CCD17815.1 |
|  | NvitOBP47 | CCD17816.1 |
|  | NvitOBP48 | CCD17817.1 |
|  | NvitOBP49 | CCD17818.1 |
|  | NvitOBP50 | CCD17819.1 |
|  | NvitOBP51 | CCD17820.1 |
|  | NvitOBP52 | CCD17821.1 |
|  | NvitOBP53 | CCD17822.1 |
|  | NvitOBP54 | CCD17823.1 |
|  | NvitOBP55 | CCD17824.1 |
|  | NvitOBP56 | CCD17825.1 |
|  | NvitOBP57 | CCD17826.1 |
|  | NvitOBP58 | CCD17827.1 |
|  | NvitOBP59 | CCD17828.1 |
|  | NvitOBP60 | CCD17829.1 |
|  | NvitOBP61 | CCD17830.1 |
|  | NvitOBP62 | CCD17831.1 |
|  | NvitOBP63 | CCD17832.1 |
|  | NvitOBP64 | CCD17833.1 |
|  | NvitOBP65 | CCD17834.1 |
|  | NvitOBP66 | CCD17835.1 |
|  | NvitOBP67 | CCD17836.1 |
|  | NvitOBP68 | CCD17837.1 |
|  | NvitOBP69 | CCD17838.1 |
|  | NvitOBP70 | CCD17839.1 |
|  | NvitOBP71 | CCD17840.1 |
|  | NvitOBP72 | CCD17841.1 |
|  | NvitOBP73 | CCD17842.1 |
|  | NvitOBP74 | CCD17843.1 |
|  | NvitOBP75 | CCD17844.1 |
|  | NvitOBP76 | CCD17845.1 |
|  | NvitOBP77 | CCD17846.1 |
|  | NvitOBP78 | CCD17847.1 |
|  | NvitOBP79 | CCD17848.1 |
|  | NvitOBP80 | CCD17849.1 |
|  | NvitOBP81 | CCD17850.1 |
|  | NvitOBP82 | CCD17851.1 |
|  | NvitOBP83 | CCD17852.1 |
|  | NvitOBP84 | CCD17853.1 |
|  | NvitOBP85 | CCD17854.1 |
|  | NvitOBP86 | CCD17855.1 |
|  | NvitOBP87 | CCD17856.1 |
|  | NvitOBP88 | CCD17857.1 |
|  | NvitOBP89 | CCD17858.1 |
|  | NvitOBP90 | CCD17859.1 |
| *Osmia cornuta* | OcorOBP1 | AGI05200.1 |
|  | OcorOBP2 | AGI05201.1 |
|  | OcorOBP3 | AGI05202.1 |
|  | OcorOBP4 | AGI05203.1 |
|  | OcorOBP5 | AGI05204.1 |
|  | OcorOBP6 | AGI05205.1 |
| *Polistes dominula* | PdomOBP1 | AAP55718.1 |
| *Pterocomma salicis* | PsalOBP1 | CAR85660.1 |
|  | PsalOBP2 | CAR85661.1 |
|  | PsalOBP4 | CAR85662.1 |
|  | PsalOBP9 | CAR85663.1 |
|  | PsalOBP10 | CAX63261.1 |
| *Rhopalosiphum padi* | RpadOBP2 | CAX63253.1 |
|  | RpadOBP3 | AHL30242.1 |
|  | RpalOBP4 | AAQ96921.1 |
|  | RpadOBP5 | CAX63254.1 |
|  | RpadOBP7 | AHL30243.1 |
|  | RpadOBP10 | CAX63255.1 |
| *Rhodinus prolixus* | RproOBP | JAA76648.1 |
|  | RproOBP2 | CAX63262.1 |
|  | RproOBP4 | CAX63263.1 |
|  | RproOBP5 | CAX63264.1 |
|  | RproOBP6 | CAX63265.1 |
|  | RproOBP19a | JAA75415.1 |
|  | RproOBP prec | JAA75160.1 |
| *Solenopsis amblychila* | SambOBP_prec | AAL51115.1 |
| *S. aurea* | SaurOBP_prec | AAL51116.1 |
| *Sitobion avenae* | SaveOBP | ACW03675.2 |
|  | SaveOBP2 | AER92705.1 |
|  | SaveOBP3 | AER92706.1 |
|  | SaveOBP4 | AER92707.1 |
|  | SaveOBP5 | AER92708.1 |
|  | SaveOBP6 | CAX63251.1 |
|  | SaveOBP8 | ACX32010.2 |
|  | SaveOBP10 | CAX63252.1 |
| *S. daguerrei* | SdagOBP_prec | AAW80693.1 |
| *S. electra* | SeleOBP_prec | AAW80692.1 |
| *Sogatella furcifera* | SfurOBP1 | AHB59655.1 |
|  | SfurOBP2 | AHB59653.1 |
|  | SfurOBP3 | AHB59656.1 |
|  | SfurOBP4 | AHB59657.1 |
|  | SfurOBP5 | AHB59658.1 |
|  | SfurOBP6 | AHB59663.1 |
|  | SfurOBP7 | AHB59659.1 |
|  | SfurOBP8 | AHB59654.1 |
|  | SfurOBP9 | AHB59660.1 |
|  | SfurOBP10 | AHB59661.1 |
|  | SfurOBP11 | AHB59662.1 |
|  | SfurOBP12 | AHJ61048.1 |
| *S. geminate* | SgemOBP_prec | AAL51131.1 |
| *S. globularia* | SgloOBP_prec | AAL51132.1 |
| *Sclerodermus guani* | SguaOBP1 | ABE68830.1 |
|  | SguaOBP2 | ABE68831.1 |
| *S. interrupta* | SintOBP | ABX25616.1 |
| *S. invicta* | SinvOBP | ACI30690.1 |
|  | SinvOBP1 | ADX94398.1 |
|  | SinvOBP2 | ADX94399.1 |
|  | SinvOBP4 | ADX94400.1 |
|  | SinvOBP5 | ADX94401.1 |
|  | SinvOBP6 | ADX94402.1 |
|  | SinvOBP7 | ADX94403.1 |
|  | SinvOBP8 | ADX94404.1 |
|  | SinvOBP9 | ADX94405.1 |
|  | SinvOBP10 | ADX94406.1 |
|  | SinvOBP11 | ADX94407.1 |
|  | SinvOBP12 | ADX94408.1 |
|  | SinvOBP13 | ADX94409.1 |
|  | SinvOBP14 | ADX94410.1 |
|  | SinvOBP15 | ADX94411.1 |
|  | SinvOBP16 | ADX94412.1 |
| *S. macdonaghi* | SmacOBP | ABX25634.1 |
| *S. megergates* | SmegOBP | ABX25630.1 |
|  | SmegOBP_prec | AAW80696.1 |
| *S. nigella* | SnigOBP_prec | AAW80682.1 |
| *S. pusillignis* | SpusOBP_prec | AAW80691.1 |
| *S. quinquecuspis* | SquiOBP | ABX25622.1 |
| *S. richteri* | SricOBP | ABX25625.1 |
| *S. saevissima* | SsaeOBP | ABX25618.1 |
| *S. substituta* | SsubOBP_prec | AAW80683.1 |
| *S. tridens* | StriOBP_prec | AAW80684.1 |
| *S. xyloni* | SxylOBP_prec | AAW80690.1 |
| *Tribolium castaneum* | TcasOBP6 | EFA04594.1 |
|  | TcasOBP7 | EFA04593.1 |
|  | TcasOBP8 | EFA04687.1 |
| *Triatoma infestans* | TinfOBP | JAC17535.1 |
|  | TinfOBP1 | JAC16578.1 |
| *Tuberolachnus salignus* | TsalOBP1 | CAR85659.1 |

**
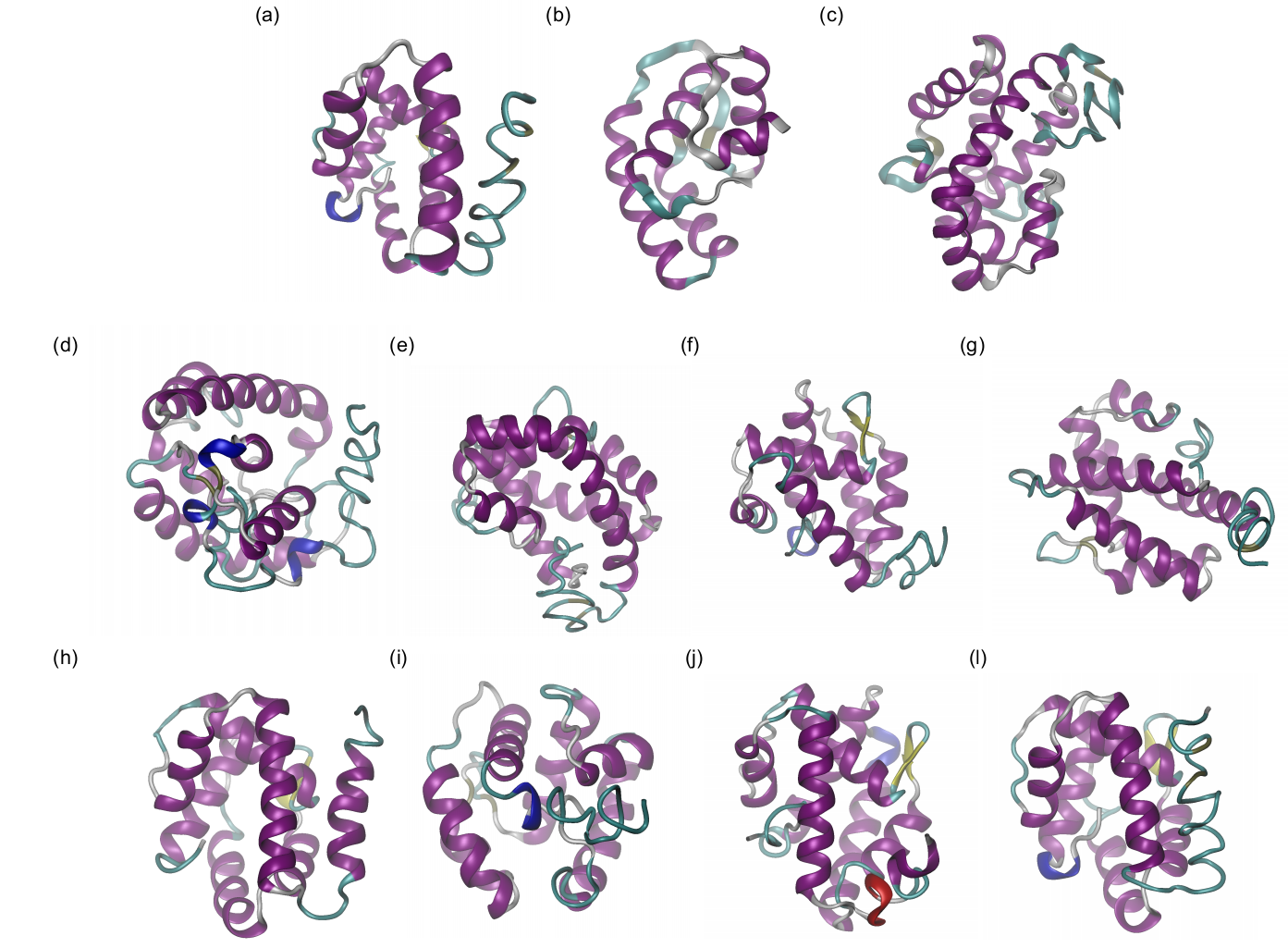
**

**Figure A. Predicted tertiary structure of the full-length putative OBPs from the stink bugs *E. heros, C. ubica* and *D. melacanthus* and from the parasitoid *T. podisi*.** (a) EherOBP3; (b) EherOBP4; (c) EherOBP5; (d) CubiOBP1; (e) CubiOBP2; (f) CubiOBP3; (g) CubiOBP4; (h) DimelOBP1; (i) TpodOBP1; (j) TpodOBP2; and (l) TpodOBP3. The structures were generated by I-TASSER server 4.2 and were oriented with N-terminus to the right side. The structures in purple are α-helixes and in red is a π-helix, in yellow are β-strands, in dark blue are turns, and in light blue and white are coils.
